# Supplementary material for: Health, lifestyle and sociodemographic characteristics are associated with Brazilian dietary patterns: Brazilian National Health Survey
Source: PLoS One. 2021 Feb 16;16(2):e0247078. doi: 10.1371/journal.pone.0247078 (PMC7886222; doi:10.1371/journal.pone.0247078)
Supplement: S1 Table — Comparison between quartile 1 and quartile 4 for each dietary pattern. (PDF) [file pone.0247078.s001.pdf]

S1 Table. Associations between dietary patterns, lifestyle, health and sociodemographic characteristics in the Southeast Region of Brazil. Comparison between quartile 1 and quartile 4 for each dietary pattern.

DIETARY PATTERNS	HEALTHY		PROTEIN		WESTEN	
**Prevalence Ratio**	**Crude (95%CI)**	**Adjusted (95%CI)**	**Crude (95%CI)**	**Adjusted (95%CI)**	**Crude (95%CI)**	**Adjusted (95%CI)**
**Sample Size (n)**	**7,140**		**6,854**		**7,403**	
**Estimated Population Size (N)**	**31,866,476**		**30,948,592**		**33,415,007**	
**Age groups (years)**						
60+	1.00	1.00	1.00	1.00	1.00	1.00
18-24	0.67(0.60-0.75)	0.62(0.55-0.69)	1.45(1.32-1.58)	1.63(1.48-1.79)	1.97(1.78-2.17)	1.58(1.42-1.76)
25-39	0.80(0.75-0.85)	0.73(0.68-0.78)	1.42(1.32-1.54)	1.53(1.41-1.66)	1.74(1.57-1.93)	1.40(1.26-1.56)
40-59	0.87(0.82-0.92)	0.83(0.78-0.87)	1.27(1.12-1.44)	1.24(1.15-1.34)	1.28(1.15-1.42)	1.12(1.01-1.25)
P-value	<0.005	<0.005	<0.005	<0.005	<0.005	<0.005
**Sex**						
Male	1.00	1.00	1.00	1.00	1.00	1.00
Female	1.23(1.16-1.29)	1.17(1.11-1.23)	0.77(0.73-0.81)	0.83(0.79-0.87)	1.03(0.98-1.09)	1.07(1.01-1.12)
P-value	<0.005	<0.005	<0.005	<0.005	0.274	0.017
**Skin Color/Race**						
White/Yellow	1.00	1.00	1.00	1.00	1.00	1.00
Others[a]	0.86(0.81-0.90)	0.93(0.88-0.98)	1.22(1.16-1.28)	1.10(1.05-1.16)	0.85(0.80-0.90)	0.90(0.85-0.95)
P-value	<0.005	0.005	<0.005	<0.005	<0.005	<0.005
**Marital status**						
Others[b]	1.00	1.00	1.00	1.00	1.00	1.00
Married	1.13(1.07-1.19)	1.09(1.03-1.15)	1.07(1.02-1.13)	1.07(1.02-1.13)	0.91(0.86-0.95)	1.02(0.97-1.07)
P-value	<0.005	<0.005	0.004	<0.005	<0.005	0.538
**Education**						
College	1.00	1.00	1.00	1.00	1.00	1.00
High School	0.83(0.78-0.88)	0.90(0.85-0.95)	1.51(1.37-1.66)	1.40(1.28-1.53)	0.88(0.83-0.92)	0.89(0.84-0.93)
Elementary School	0.81(0.76-0.87)	0.80(0.75-0.85)	1.61(1.46-1.78)	1.65(1.50-1.82)	0.58(0.54-0.62)	0.71(0.66-0.76)
Illiterate	0.68(0.59-0.79)	0.64(0.55-0.75)	1.37(1.19-1.58)	1.57(1.36-1.81)	0.41(0.34-0.50)	0.57(0.47-0.69)
P-value	<0.005	<0.005	<0.005	<0.005	<0.005	<0.005
**Area of residence**						
Urban area	1.00	-	1.00	1.00	1.00	1.00
Rural area	0.82(0.73-0.92)	-	1.32(1.25-1.39)	1.16(1.10-1.23)	0.60(0.51-0.70)	0.70(0.61-0.81)
P-value	<0.005	-	<0.005	<0.005	<0.005	<0.005
**Economic Status**						
A-B	1.00	-	1.00	-	1.00	-

C	0.90(0.84-0.96)	-	1.11(1.04-1.18)	-	0.91(0.85-0.97)	-
D-E	0.88(0.82-0.94)	-	1.13(1.06-1.21)	-	0.85(0.79-0.92)	-
P-value	<0.005	-	<0.005	-	<0.005	-
**Physical Activity**						
Sufficient	1.00	1.00	1.00	1.00	1.00	-
Insufficient	0.91(0.84-0.98)	0.87(0.81-0.94)	1.06(0.99-1.13)	1.13(1.06-1.20)	0.95(0.88-1.03)	-
None	0.87(0.82-0.93)	0.83(0.78-0.89)	0.97(0.91-1.03)	1.05(0.99-1.12)	0.90(0.85-0.96)	-
P-value	<0.005	<0.005	0.044	<0.005	0.005	-
**Smoking**						
Never	1.00	1.00	1.00	1.00	1.00	1.00
Ex-smokers	0.96(0.90-1.03)	0.94(0.88-1.01)	1.04(0.98-1.11)	1.02(0.96-1.09)	0.82(0.76-0.90)	0.98(0.91-1.06)
Current	0.76(0.70-0.82)	0.83(0.76-0.90)	1.23(1.17-1.30)	1.11(1.05-1.17)	0.93(0.87-1.00)	1.02(0.95-1.09)
P-value	<0.005	<0.005	<0.005	<0.005	<0.005	<0.005
**Alcohol intake**						
Abstainer	1.00	1.00	1.00	1.00	1.00	1.00
Moderate	0.96(0.91-1.02)	0.96(0.91-1.02)	1.08(1.02-1.16)	1.06(1.00-1.13)	1.20(1.13-1.28)	1.07(1.01-1.14)
Binge drinker	0.77(0.69-0.85)	0.86(0.78-0.95)	1.25(1.18-1.33)	1.11(1.04-1.18)	1.26(1.18-1.34)	1.12(1.05-1.19)
P-value	<0.005	<0.015	<0.005	<0.005	<0.005	<0.005
**Self-Rated Health**						
Very good/Good	1.00	-	1.00	-	1.00	1.00
Fair	0.97(0.91-1.03)	-	0.99(0.93-1.05)	-	0.71(0.66-0.77)	0.89(0.83-0.95)
Poor/Very poor	0.90(0.80-1.02)	-	0.99(0.89-1.11)	-	0.60(0.50-0.71)	0.83(0.70-0.98)
P-value	0.219	-	0.912	-	<0.005	<0.005
**Multimorbidity**						
0 or 1	1.00	-	1.00	-	1.00	-
2	1.10(1.02-1.19)	-	0.83(0.76-0.91)	-	0.84(0.77-0.92)	-
3	1.18(1.10-1.28)	-	0.79(0.70-0.90)	-	0.73(0.64-0.84)	-
4+	1.19(1.11-1.28)	-	0.81(0.72-0.92)	-	0.65(0.55-0.76)	-
P-value	<0.005	-	<0.005	-	<0.005	-

P-value to the Wald Test.
-: Variables not statistically significant in the model.
[a] Black(a), brown(a), indigenous.
[b] single, divorced, separated, widowed
