# Supplementary material for: Health, lifestyle and sociodemographic characteristics are associated with Brazilian dietary patterns: Brazilian National Health Survey
Source: PLoS One. 2021 Feb 16;16(2):e0247078. doi: 10.1371/journal.pone.0247078 (PMC7886222; doi:10.1371/journal.pone.0247078)
Supplement: S2 Table — Comparison between quartile 1 and quartile 4 for each dietary pattern. (PDF) [file pone.0247078.s002.pdf]

S2 Table. Associations between dietary patterns, lifestyle, health and sociodemographic characteristics in the South Region of Brazil. Comparison between quartile 1 and quartile 4 for each dietary pattern.

**DIETARY PATTERNS**	HEALTHY		PROTEIN		WESTEN	
**Prevalence Ratio**	**Crude (95%CI)**	**Adjusted (95%CI)**	**Crude (95%CI)**	**Adjusted (95%CI)**	**Crude (95%CI)**	**Adjusted (95%CI)**
**Sample Size (n)**	**3,632**		**3,484**		**3,948**	
**Estimated Population Size (N)**	**10,185,306**		**10,111,326**		**11,255,746**	
**Age groups (years)**						
60+	1.00	1.00	1.00	1.00	1.00	1.00
18-24	0.54(0.46-0.63)	0.49(0.41-0.58)	1.36(1.19-1.57)	1.53(1.32-1.76)	1.77(1.59-1.97)	1.52(1.37-1.69)
25-39	0.72(0.64-0.81)	0.63(0.56-0.71)	1.26(1.12-1.43)	1.39(1.22-1.58)	1.59(1.43-1.77)	1.38(1.24-1.54)
40-59	0.89(0.81-0.99)	0.84(0.77-0.93)	1.27(1.12-1.44)	1.27(1.12-1.45)	1.27(1.13-1.43)	1.19(1.06-1.32)
P-value	<0.005	<0.005	<0.005	<0.005	<0.005	<0.005
**Sex**						
Male	1.00	1.00	1.00	1.00	1.00	1.00
Female	1.43(1.31-1.56)	1.31(1.21-1.43)	0.74(0.68-0.79)	0.76(0.71-0.81)	1.04(0.97-1.11)	1.06(1.00-1.13)
P-value	<0.005	<0.005	<0.005	0.254	0.274	0.037
**Skin Color/Race**						
White/Yellow	1.00	1.00	1.00	1.00	1.00	1.00
Others[a]	0.75(0.67-0.84)	0.85(0.77-0.95)	1.27(1.17-1.37)	1.18(1.09-1.27)	0.79(0.73-0.87)	0.83(0.76-0.91)
P-value	<0.005	0.006	<0.005	<0.005	<0.005	<0.005
**Marital status**						
Others[b]	1.00	-	1.00	1.00	1.00	-
Married	1.13(1.04-1.23)	-	1.18(1.08-1.29)	1.12(1.03-1.22)	0.95(0.90-1.01)	-
P-value	<0.005	-	<0.005	0.008	0.125	-
**Education**						
College	1.00	1.00	1.00	1.00	1.00	1.00
High School	0.85(0.77-0.94)	0.88(0.80-0.97)	1.51(1.32-1.72)	1.43(1.27-1.61)	0.97(0.93-1.02)	1.00(0.96-1.05)
Elementary School	0.76(0.68-0.86)	0.70(0.62-0.78)	1.60(1.41-1.81)	1.59(1.42-1.77)	0.66(0.61-0.71)	0.80(0.74-0.87)
Illiterate	0.81(0.66-1.00)	0.65(0.53-0.81)	1.36(1.13-1.64)	1.58(1.30-1.91)	0.53(0.43-0.66)	0.72(0.58-0.90)
P-value	<0.005	<0.005	<0.005	<0.005	<0.005	<0.005
**Area of residence**						
Urban area	1.00	1.00	1.00	-	1.00	1.00
Rural area	1.07(0.97-1.18)	1.15(1.04-1.27)	1.19(1.09-1.31)	-	0.71(0.61-0.83)	0.79(0.68-0.92)
P-value	0.155	<0.005	<0.005	-	<0.005	<0.005
**Economic Status**						
A-B	1.00	-	1.00	-	1.00	-
C	0,91(0,82 - 1,01)	-	1,12(1,04 - 1,21)	-	0,90(0,84 - 0,96)	-
D-E	0,86(0,76 - 0,96)	-	1,06(0,96 - 1,16)	-	0,85(0,79 - 0,92)	-
P-value	0.026	-	0.012	-	<0.005	-

**Physical Activity**						
Sufficient	1.00	1.00	1.00	1.00	1.00	-
Insufficient	0,90(0,79 - 1,02)	0,86(0,77 - 0,97)	1,02(0,92 - 1,15)	1,06(0,96 - 1,17)	1,00(0,92 - 1,07)	-
None	0,93(0,84 - 1,03)	0,88(0,80 - 0,97)	1,07(0,98 - 1,16)	1,13(1,04 - 1,22)	0,91(0,85 - 0,98)	-
P-value	0.145	0.005	0.297	0.014	0.033	-
**Smoking**						
Never	1.00	1.00	1.00	1.00	1.00	1.00
Ex-smokers	0,95(0,85 - 1,07)	0,99(0,90 - 1,10)	1,07(0,97 - 1,18)	1,03(0,95 - 1,13)	0,91(0,85 - 0,97)	1,03(0,96 - 1,10)
Current	0,60(0,50 - 0,72)	0,72(0,60 - 0,86)	1,30(1,21 - 1,40)	1,17(1,09 - 1,26)	0,80(0,73 - 0,87)	0,89(0,82 - 0,97)
P-value	<0.005	<0.005	<0.05	<0.005	<0.005	0.011
**Alcohol intake**						
Abstainer	1.00	1.00	1.00	-	1.00	1.00
Moderate	0,89(0,81 - 0,97)	0,93(0,86 - 1,02)	1,04(0,97 - 1,12)	-	1,17(1,11 - 1,23)	1,08(1,02 - 1,14)
Binge drinker	0,51(0,42 - 0,62)	0,63(0,53 - 0,76)	1,16(1,03 - 1,31)	-	1,17(1,05 - 1,30)	1,04(0,95 - 1,15)
P-value	<0.005	<0.005	0.048	-	<0.005	0.023
**Self-Rated Health**						
Very good/Good	1.00	1.00	1.00	-	1.00	-
Fair	0,95(0,86 - 1,05)	0,88(0,80 - 0,97)	1,01(0,93 - 1,11)	-	0,75(0,68 - 0,83)	-
Poor/Very poor	0,83(0,66 - 1,05)	0,76(0,60 - 0,97)	0,95(0,80 - 1,12)	-	0,64(0,53 - 0,77)	-
P-value	0.199	0.007	0.761	-	<0.005	-
**Multimorbidity**						
0 or 1	1.00	-	1.00	-	1.00	-
2	1,11(0,99 - 1,25)	-	0,95(0,86 - 1,05)	-	0,84(0,77 - 0,92)	-
3	1,32(1,17 - 1,49)	-	0,83(0,71 - 0,97)	-	0,77(0,67 - 0,89)	-
4+	1,21(1,06 - 1,38)	-	0,70(0,58 - 0,84)	-	0,72(0,62 - 0,84)	-
P-value	<0.005	-	<0.005	-	<0.005	-

P-value to the Wald Test.

-: Variables not statistically significant in the model.

[a] Black(a), brown(a), indigenous.

[b] single, divorced, separated, widowed
