# Supplementary material for: Health, lifestyle and sociodemographic characteristics are associated with Brazilian dietary patterns: Brazilian National Health Survey
Source: PLoS One. 2021 Feb 16;16(2):e0247078. doi: 10.1371/journal.pone.0247078 (PMC7886222; doi:10.1371/journal.pone.0247078)
Supplement: S3 Table — Comparison between quartile 1 and quartile 4 for each dietary pattern. (PDF) [file pone.0247078.s003.pdf]

S3 Table. Prevalence Ratio (PR) and confidence interval (95%CI) for associations between dietary patterns, lifestyle, health and sociodemographic characteristics in the Midwest Region of Brazil. Poisson regression models. Comparison between quartile 1 and quartile 4 for each dietary pattern

DIETARY PATTERNS	HEALTHY		PROTEIN		WESTEN	
**Prevalence Ratio**	**Crude (95%CI)**	**Adjusted (95%CI)**	**Crude (95%CI)**	**Adjusted (95%CI)**	**Crude (95%CI)**	**Adjusted (95%CI)**
**Sample Size (n)**	**3,841**		**4,326**		**3,743**	
**Estimated Population Size (N)**	**5,316,604**		**6,375,641**		**5,328,569**	
**Age groups (years)**						
60+	1.00	1.00	1.00	1.00	1.00	1.00
18-24	0.66(0.58-0.77)	0.54(0.46-0.62)	1.16(1.10-1.22)	1.27(1.20-1.35)	2.29(1.96-2.66)	1.67(1.43-1.96)
25-39	0.83(0.76-0.91)	0.71(0.64-0.78)	1.09(1.03-1.15)	1.16(1.09-1.23)	1.82(1.56-2.13)	1.39(1.19-1.63)
40-59	0.94(0.86-1.02)	0.86(0.79-0.93)	1.27(1.12-1.44)	1.09(1.03-1.16)	1.24(1.06-1.45)	1.08(0.93-1.26)
P-value	<0.005	<0.005	<0.005	<0.005	<0.005	<0.005
**Sex**						
Male	1.00	1.00	1.00	1.00	1.00	-
Female	1.41(1.31-1.52)	1.34(1.24-1.43)	0.89(0.86-0.92)	0.91(0.88-0.94)	0.95(0.89-1.03)	-
P-value	<0.005	<0.005	<0.005	<0.005	0.022	-
**Skin Color/Race**						
White/Yellow	1.00	1.00	1.00	-	1.00	1.00
Others[a]	0.82(0.76-0.87)	0.89(0.84-0.95)	1.06(1.02-1.10)	-	0.92(0.86-0.99)	0.94(0.88-1.00)
P-value	<0.005	<0.005	<0.005	-	0.019	<0.005
**Marital status**						
Others[b]	1.00	-	1.00	1.00	1.00	-
Married	1.09(1.02-1.16)	-	1.07(1.04-1.11)	1.07(1.03-1.11)	0.82(0.77-0.88)	-
P-value	0.015	-	<0.005	<0.005	<0.005	-
**Education**						
College	1.00	1.00	1.00	1.00	1.00	1.00
High School	0.81(0.75-0.87)	0.90(0.84-0.97)	1.20(1.13-1.27)	1.16(1.10-1.23)	0.95(0.89-1.02)	0.97(0.91-1.03)
Elementary School	0.69(0.63-0.75)	0.76(0.69-0.82)	1.24(1.17-1.32)	1.24(1.17-1.31)	0.59(0.54-0.65)	0.77(0.70-0.85)
Illiterate	0.68(0.60-0.78)	0.69(0.60-0.80)	1.21(1.13-1.30)	1.29(1.19-1.39)	0.47(0.39-0.58)	0.75(0.61-0.92)
P-value	<0.005	<0.005	<0.005	<0.005	<0.005	<0.005
**Area of residence**						
Urban area	1.00	-	1.00	-	1.00	1.00
Rural area	0.84(0.74-0.95)	-	1.09(1.05-1.13)	-	0.53(0.46-0.62)	0.63(0.54-0.72)
P-value	0.008	-	<0.005	-	<0.005	<0.005
**Economic Status**						
A-B	1.00	1.00	1.00	1.00	1.00	1.00
C	0.78(0.71-0.84)	0.86(0.80-0.93)	1.12(1.07-1.17)	1.08(1.04-1.13)	0.85(0.78-0.92)	0.95(0.89-1.02)
D-E	0.79(0.73-0.86)	0.91(0.84-0.98)	1.14(1.08-1.19)	1.09(1.04-1.14)	0.78(0.72-0.85)	0.92(0.86-1.00)

P-value	<0.005	<0.005	<0.005	<0.005	<0.005	<0.005
**Physical Activity**						
Sufficient	1.00	1.00	1.00	1.00	1.00	-
Insufficient	0.93(0.85-1.02)	0.90(0.84-0.98)	1.04(0.99-1.08)	1.04(1.00-1.08)	1.03(0.94-1.13)	-
None	0.91(0.84-1.00)	0.88(0.81-0.95)	1.06(1.02-1.10)	1.08(1.04-1.12)	0.83(0.76-0.91)	-
P-value	0.057	<0.005	0.006	<0.005	<0.005	-
**Smoking**						
Never	1.00	1.00	1.00	-	1.00	-
Ex-smokers	0.91(0.83-1.00)	0.92(0.85-1.00)	1.03(0.99-1.07)	-	0.76(0.68-0.84)	-
Current	0.67(0.59-0.77)	0.77(0.68-0.87)	1.08(1.04-1.13)	-	0.78(0.69-0.88)	-
P-value	<0.005	<0.005	<0.005	-	<0.005	-
**Alcohol intake**						
Abstainer	1.00	1.00	1.00	1.00	1.00	1.00
Moderate	1.04(0.98-1.11)	1.08(1.02-1.15)	1.02(0.98-1.06)	1.03(0.99-1.07)	1.18(1.08-1.29)	1.06(0.98-1.15)
Binge drinker	0.76(0.67-0.86)	0.90(0.80-1.01)	1.10(1.06-1.14)	1.06(1.02-1.10)	1.31(1.20-1.43)	1.10(1.02-1.19)
P-value	<0.005	<0.005	<0.005	0.021	<0.005	0.033
**Self-Rated Health**						
Very good/Good	1.00	1.00	1.00	-	1.00	1.00
Fair	0.93(0.86-1.00)	0.93(0.86-1.00)	1.01(0.98-1.04)	-	0.72(0.66-0.79)	0.92(0.84-1.01)
Poor/Very poor	0.76(0.63-0.91)	0.75(0.63-0.89)	0.97(0.90-1.05)	-	0.46(0.36-0.59)	0.70(0.54-0.91)
P-value	<0.005	<0.005	0.657	-	<0.005	0.010
**Multimorbidity**						
0 or 1	1.00	-	1.00	-	1.00	1.00
2	1.02(0.92-1.15)	-	0.99(0.94-1.04)	-	0.70(0.61-0.80)	0.85(0.75-0.97)
3	1.15(1.01-1.30)	-	0.93(0.85-1.01)	-	0.56(0.45-0.69)	0.77(0.62-0.96)
4+	1.17(1.03-1.33)	-	0.90(0.82-0.98)	-	0.60(0.46-0.79)	0.93(0.71-1.22)
P-value	0.027	-	0.034	-	<0.005	0.015

P-value to the Wald Test.

-: Variables not statistically significant in the model.

[a] Black(a), brown(a), indigenous.

[b] single, divorced, separated, widowed
