# Supplementary material for: Health, lifestyle and sociodemographic characteristics are associated with Brazilian dietary patterns: Brazilian National Health Survey
Source: PLoS One. 2021 Feb 16;16(2):e0247078. doi: 10.1371/journal.pone.0247078 (PMC7886222; doi:10.1371/journal.pone.0247078)
Supplement: S4 Table — Comparison between quartile 1 and quartile 4 for each dietary pattern. (PDF) [file pone.0247078.s004.pdf]

S4 Table. Associations between dietary patterns, lifestyle, health and sociodemographic characteristics in the Northeast Region of Brazil. Comparison between quartile 1 and quartile 4 for each dietary pattern.

DIETARY PATTERNS	HEALTHY		PROTEIN		WESTEN	
**Prevalence Ratio**	**Crude (95%CI)**	**Adjusted (95%CI)**	**Crude (95%CI)**	**Adjusted (95%CI)**	**Crude (95%CI)**	**Adjusted (95%CI)**
**Sample Size (n)**	**8,775**		**8,546**		**9,385**	
**Estimated Population Size (N)**	**18,942,835**		**18,546,789**		**20,219,444**	
**Age groups (years)**						
60+	1.00	1.00	1.00	1.00	1.00	1.00
18-24	0.56(0.48-0.66)	0.36(0.31-0.42)	1.41(1.19-1.67)	1.51(1.26-1.81)	5.27(4.40-6.31)	3.37(2.80-4.06)
25-39	0.79(0.71-0.89)	0.54(0.48-0.61)	1.32(1.15-1.51)	1.38(1.19-1.60)	3.19(2.64-3.84)	2.18(1.81-2.63)
40-59	0.83(0.73-0.93)	0.68(0.61-0.76)	1.27(1.12-1.44)	1.20(1.04-1.38)	1.74(1.42-2.13)	1.36(1.11-1.65)
P-value	<0.005	<0.005	<0.005	<0.005	<0.005	<0.005
**Sex**						
Male	1.00	1.00	1.00	1.00	1.00	-
Female	1.38(1.25-1.51)	1.23(1.13-1.33)	0.57(0.52-0.62)	0.64(0.58-0.70)	1.01(0.90-1.12)	-
P-value	<0.005	<0.005	<0.005	<0.005		-
**Skin Color/Race**						
White/Yellow	1.00	-	1.00	-	1.00	-
Others[a]	0.82(0.76-0.89)	-	1.06(0.95-1.18)	-	0.90(0.81-1.00)	-
P-value	<0.005	-	0.311	-	<0.005	-
**Marital status**						
Others[b]	1.00	1.00	1.00	-	1.00	-
Married	1.09(1.00-1.19)	1.11(1.02-1.21)	1.10(0.99-1.22)	-	0.70(0.64-0.77)	-
P-value	0.06	0.02	0.08	-	<0.005	-
**Education**						
College	1.00	1.00	1.00	1.00	1.00	1.00
High School	0.77(0.69-0.85)	0.88(0.80-0.97)	1.29(1.10-1.51)	1.20(1.03-1.40)	0.94(0.85-1.04)	0.90(0.82-0.99)
Elementary School	0.56(0.50-0.63)	0.66(0.59-0.73)	1.58(1.36-1.83)	1.40(1.22-1.62)	0.50(0.44-0.56)	0.67(0.60-0.75)
Illiterate	0.38(0.32-0.45)	0.43(0.35-0.51)	1.54(1.31-1.82)	1.54(1.29-1.83)	0.22(0.17-0.28)	0.44(0.35-0.57)
P-value	<0.005	<0.005	<0.005	<0.005	<0.005	<0.005
**Area of residence**						
Urban area	1.00	1.00	1.00	1.00	1.00	1.00
Rural area	0.54(0.46-0.63)	0.71(0.61-0.83)	1.56(1.42-1.71)	1.40(1.26-1.55)	0.47(0.39-0.56)	0.59(0.50-0.71)
P-value	<0.005	<0.005	<0.005	<0.005	<0.005	<0.005
**Economic Status**						
A-B	1.00	1.00	1.00	-	1.00	1.00
C	0.90(0.78-1.03)	1.06(0.95-1.18)	1.21(1.01-1.44)	-	0.70(0.61-0.81)	0.81(0.74-0.90)
D-E	0.60(0.52-0.70)	0.89(0.78-1.00)	1.36(1.17-1.59)	-	0.56(0.49-0.64)	0.87(0.77-0.98)
P-value	<0.005	<0.005	<0.005	-	<0.005	<0.005

**Physical Activity**						
Sufficient	1.00	1.00	1.00	-	1.00	-
Insufficient	0.86(0.76-0.97)	0.85(0.76-0.96)	0.95(0.85-1.05)	-	0.95(0.84-1.07)	-
None	0.79(0.72-0.88)	0.80(0.73-0.88)	0.96(0.86-1.07)	-	0.72(0.65-0.81)	-
P-value	<0.005	<0.005	0.54	-	<0.005	-
**Smoking**						
Never	1.00	1.00	1.00	1.00	1.00	-
Ex-smokers	0.84(0.75-0.94)	0.82(0.74-0.91)	1.10(1.00-1.21)	1.09(0.99-1.21)	0.57(0.50-0.66)	-
Current	0.49(0.42-0.57)	0.57(0.49-0.66)	1.45(1.33-1.59)	1.23(1.13-1.35)	0.75(0.64-0.86)	-
P-value	<0.005	<0.05	<0.05	<0.005	<0.005	-
**Alcohol intake**						
Abstainer	1.00	-	1.00	1.00	1.00	1.00
Moderate	0.88(0.80-0.97)	-	1.17(1.04-1.33)	1.00(0.89-1.12)	1.57(1.40-1.76)	1.26(1.14-1.39)
Binge drinker	0.77(0.68-0.88)	-	1.51(1.35-1.68)	1.15(1.03-1.29)	1.60(1.43-1.79)	1.25(1.14-1.38)
P-value	<0.005	<0.005	<0.005	0.022	<0.005	<0.005
**Self-Rated Health**						
Very good/Good	1.00	1.00	1.00	-	1.00	-
Fair	0.79(0.72-0.86)	0.76(0.69-0.84)	0.93(0.84-1.03)	-	0.66(0.59-0.75)	-
Poor/Very poor	0.69(0.58-0.82)	0.70(0.59-0.84)	0.86(0.74-0.99)	-	0.43(0.35-0.53)	-
P-value	<0.005	<0.005	0.08	-	<0.005	-
**Multimorbidity**						
0 or 1	1.00	1.00	1.00	1.00	1.00	-
2	1.17(1.03-1.33)	1.10(0.99-1.22)	0.77(0.68-0.87)	0.90(0.79-1.02)	0.58(0.49-0.68)	-
3	1.35(1.14-1.59)	1.24(1.07-1.43)	0.75(0.63-0.90)	0.92(0.75-1.12)	0.50(0.36-0.69)	-
4+	1.54(1.35-1.76)	1.33(1.17-1.52)	0.44(0.33-0.59)	0.58(0.43-0.78)	0.43(0.33-0.56)	-
P-value	<0.005	<0.005	<0.005	<0.005	<0.005	-

P-value to the Wald Test.

-: Variables not statistically significant in the model.

[a] Black(a), brown(a), indigenous.

[b] single, divorced, separated, widowed
