# Supplementary material for: Health, lifestyle and sociodemographic characteristics are associated with Brazilian dietary patterns: Brazilian National Health Survey
Source: PLoS One. 2021 Feb 16;16(2):e0247078. doi: 10.1371/journal.pone.0247078 (PMC7886222; doi:10.1371/journal.pone.0247078)
Supplement: S5 Table — Comparison between quartile 1 and quartile 4 for each dietary pattern. (PDF) [file pone.0247078.s005.pdf]

S5 Table. Associations between dietary patterns, lifestyle, health and sociodemographic characteristics in the North Region of Brazil. Comparison between quartile 1 and quartile 4 for each dietary pattern.

DIETARY PATTERNS	HEALTHY		PROTEIN		WESTEN	
**Prevalence Ratio**	**Crude (95%CI)**	**Adjusted (95%CI)**	**Crude (95%CI)**	**Adjusted (95%CI)**	**Crude (95%CI)**	**Adjusted (95%CI)**
**Sample Size (n)**	**6,713**		**6,891**		**5,649**	
**Estimated Population Size (N)**	**5,879,138**		**6,153,627**		**4,988,943**	
**Age groups (years)**						
60+	1.00	1.00	1.00	1.00	1.00	1.00
18-24	0.67(0.50-0.89)	0.45(0.33-0.61)	1.75(1.35-2.26)	1.90(1.47-2.44)	5.09(3.41-7.58)	3.58(2.44-5.25)
25-39	0.81(0.65-1.00)	0.55(0.42-0.71)	1.70(1.32-2.17)	1.69(1.32-2.16)	3.56(2.39-5.29)	2.80(1.92-4.09)
40-59	1.10(0.89-1.36)	0.88(0.71-1.09)	1.27(1.12-1.44)	1.32(1.01-1.72)	1.85(1.22-2.80)	1.66(1.13-2.44)
P-value	<0.005	<0.005	<0.005	<0.005	<0.005	<0.005
**Sex**						
Male	1.00	1.00	1.00	1.00	1.00	-
Female	1.52(1.31-1.78)	1.39(1.21-1.60)	0.81(0.71-0.91)	0.81(0.72-0.91)	0.92(0.83-1.03)	-
P-value	<0.005	<0.005	<0.005	<0.005	0.17	-
**Skin Color/Race**						
White/Yellow	1.00	1.00	1.00	1.00	1.00	1.00
Others[a]	0.61(0.51-0.72)	0.76(0.65-0.89)	0.73(0.63-0.85)	0.71(0.62-0.82)	0.77(0.68-0.87)	0.88(0.78-0.98)
P-value	<0.005	<0.005	<0.005	<0.005	<0.005	0.02
**Marital status**						
Others[b]	1.00	-	1.00	1.00	1.00	-
Married	1.04(0.88-1.23)	-	1.17(1.02-1.35)	1.27(1.10-1.45)	0.73(0.65-0.82)	-
P-value	0.62	-		<0.005	<0.005	-
**Education**						
College	1.00	1.00	1.00	-	1.00	1.00
High School	0.53(0.45-0.62)	0.63(0.54-0.72)	1.15(0.92-1.44)	-	0.90(0.79-1.03)	0.93(0.82-1.05)
Elementary School	0.37(0.30-0.45)	0.45(0.37-0.54)	1.04(0.84-1.30)	-	0.34(0.29-0.41)	0.59(0.49-0.72)
Illiterate	0.40(0.29-0.54)	0.44(0.30-0.65)	0.94(0.72-1.23)	-	0.31(0.21-0.46)	0.62(0.41-0.92)
P-value	<0.005	<0.005	<0.005	-	<0.005	<0.005
**Area of residence**						
Urban area	1.00	1.00	1.00	1.00	1.00	1.00
Rural area	0.37(0.27-0.49)	0.49(0.37-0.66)	0.75(0.59-0.94)	0.70(0.55-0.89)	0.22(0.16-0.30)	0.29(0.21-0.40)
P-value	<0.005	<0.005	<0.005	<0.005	<0.005	<0.005
**Economic Status**						
A-B	1.00	-	1.00	1.00	1.00	1.00
C	0.92(0.77-1.10)	-	1.15(0.94-1.41)	1.21(1.00-1.46)	0.75(0.66-0.85)	0.89(0.79-1.00)
D-E	0.63(0.51-0.77)	-	1.16(0.95-1.41)	1.33(1.10-1.62)	0.53(0.46-0.61)	0.85(0.75-0.96)
P-value	<0.005	-	0.32	<0.005	<0.005	<0.005

**Physical Activity**						
Sufficient	1.00	1.00	1.00	-	1.00	-
Insufficient	0.84(0.68-1.03)	0.79(0.65-0.95)	0.93(0.77-1.13)	-	0.81(0.69-0.96)	-
None	0.75(0.63-0.89)	0.73(0.61-0.87)	0.94(0.80-1.10)	-	0.71(0.62-0.82)	-
P-value	<0.005	<0.005	0.63	<0.005	<0.005	-
**Smoking**						
Never	1.00	-	1.00	1.00	1.00	-
Ex-smokers	0.94(0.77-1.15)	-	1.01(0.82-1.23)	1.12(0.93-1.36)	0.67(0.55-0.81)	-
Current	0.68(0.53-0.88)	-	1.37(1.17-1.61)	1.36(1.16-1.59)	0.80(0.66-0.98)	-
P-value	0.013	-	<0.005		<0.05	-
**Alcohol intake**						
Abstainer	1.00	-	1.00	-	1.00	-
Moderate	0.90(0.76-1.07)	-	1.23(1.07-1.41)	-	1.26(1.11-1.44)	-
Binge drinker	0.80(0.61-1.03)	-	1.39(1.17-1.67)	-	1.59(1.37-1.85)	-
P-value	0.133	-	<0.005	<0.005	<0.005	-
**Self-Rated Health**						
Very good/Good	1.00	1.00	1.00	-	1.00	-
Fair	0.74(0.61-0.90)	0.75(0.64-0.89)	0.91(0.78-1.06)	-	0.69(0.60-0.80)	-
Poor/Very poor	0.66(0.48-0.91)	0.65(0.47-0.90)	0.85(0.64-1.13)	-	0.38(0.28-0.52)	-
P-value	0.015	<0.005	0.322	<0.005	<0.005	-
**Multimorbidity**						
0 or 1	1.00	1.00	1.00	-	1.00	-
2	1.25(1.00-1.57)	1.11(0.89-1.40)	1.10(0.90-1.33)	-	0.71(0.58-0.87)	-
3	1.70(1.35-2.15)	1.48(1.20-1.82)	0.85(0.65-1.12)	-	0.68(0.52-0.91)	-
4+	1.51(1.06-2.16)	1.25(0.89-1.74)	0.71(0.48-1.06)	-	0.41(0.26-0.65)	-
P-value	<0.005	<0.005	0.124	<0.005	<0.005	-

P-value to the Wald Test.

-: Variables not statistically significant in the model.

[a] Black(a), brown(a), indigenous.

[b] single, divorced, separated, widowed
