# Supplementary material for: Health, lifestyle and sociodemographic characteristics are associated with Brazilian dietary patterns: Brazilian National Health Survey
Source: PLoS One. 2021 Feb 16;16(2):e0247078. doi: 10.1371/journal.pone.0247078 (PMC7886222; doi:10.1371/journal.pone.0247078)
Supplement: S6 Table — Comparison between quartile 1 and quartile 2 for each dietary pattern. (PDF) [file pone.0247078.s006.pdf]

S6 Table. Associations between dietary patterns, lifestyle, health and sociodemographic characteristics in Brazil. Comparison between quartile 1 and quartile 2 for each dietary pattern

**DIETARY PATTERNS**	**HEALTHY**		**PROTEIN**		**WESTEN**	
**Prevalence Ratio**	**Crude (95%CI)**	**Adjusted (95%CI)**	**Crude (95%CI)**	**Adjusted (95%CI)**	**Crude (95%CI)**	**Adjusted (95%CI)**
**Sample Size (n)**	**30,101**		**30,101**		**30,101**	
**Estimated Population Size (N)**	**69,093,963**		**64,559,971**		**68,239,229**	
**Age groups (years)**						
60+	1.00	1.00	1.00	1.00	1.00	1.00
18-24	0.83(0.77-0.89)	0.71(0.65-0.77)	1.11(1.04-1.18)	1.17(1.09-1.26)	1.46(1.36-1.56)	1.28(1.19-1.38)
25-39	0.95(0.90-1.00)	0.82(0.77-0.87)	1.11(1.05-1.16)	1.14(1.08-1.21)	1.30(1.23-1.38)	1.17(1.10-1.25)
40-59	0.96(0.91-1.01)	0.88(0.83-0.93)	1.08(1.02-1.15)	1.09(1.03-1.15)	1.11(1.05-1.18)	1.05(0.99-1.12)
P-value	<0.005	<0.005	<0.005	<0.005	<0.005	<0.005
**Sex**						
Male	1.00	1.00	1.00	1.00	1.00	-
Female	1.10(1.06-1.14)	1.08(1.04-1.12)	0.86(0.83-0.89)	0.87(0.84-0.90)	1.00(0.96-1.04)	-
P-value	<0.005	<0.005	<0.005	<0.005	0.859	-
**Skin Color/Race**						
White/Yellow	1.00	1.00	1.00	-	1.00	-
Others[a]	0.83(0.80-0.87)	0.92(0.88-0.96)	0.92(0.89-0.96)	-	0.91(0.88-0.95)	-
P-value	<0.005	<0.005	<0.005	-	<0.005	-
**Marital status**						
Others[b]	1.00	1.00	1.00	1.00	1.00	-
Married	1.06(1.02-1.11)	1.06(1.02-1.11)	1.07(1.03-1.11)	1.07(1.03-1.11)	1.00(0.96-1.04)	-
P-value	<0.005	<0.005	<0.005	<0.005	0.924	-
**Education**						
College	1.00	1.00	1.00	1.00	1.00	1.00
High School	0.90(0.86-0.95)	0.95(0.90-1.00)	1.13(1.07-1.19)	1.17(1.11-1.23)	0.93(0.88-0.98)	0.94(0.90-0.99)
Elementary School	0.82(0.78-0.87)	0.85(0.80-0.90)	1.09(1.04-1.15)	1.18(1.11-1.24)	0.75(0.71-0.79)	0.84(0.80-0.89)
Illiterate	0.69(0.64-0.75)	0.72(0.66-0.79)	0.99(0.91-1.07)	1.15(1.06-1.24)	0.60(0.55-0.65)	0.77(0.70-0.84)
P-value	<0.005	<0.005	<0.005	<0.005	<0.005	<0.005
**Area of residence**						
Urban area	1.00	1.00	1.00	-	1.00	1.00
Rural area	0.79(0.74-0.84)	0.88(0.83-0.94)	0.95(0.89-1.00)	-	0.78(0.73-0.83)	0.84(0.79-0.89)
P-value	<0.005	<0.005	0.070	-	<0.005	<0.005
**Economic Status**						

A-B	1.00	-	1.00	-	1.00	-
C	0.90(0.85-0.95)	-	0.99(0.94-1.03)	-	0.91(0.85-0.96)	-
D-E	0.80(0.76-0.85)	-	0.92(0.88-0.97)	-	0.80(0.76-0.85)	-
P-value	<0.005	-	<0.005	-	<0.005	-
**Physical Activity**						
Sufficient	1.00	1.00	1.00	1.00	1.00	-
Insufficient	0.99(0.94-1.04)	0.96(0.92-1.01)	1.04(0.99-1.09)	1.06(1.01-1.11)	0.94(0.89-1.00)	-
None	0.96(0.91-1.00)	0.93(0.89-0.97)	1.04(0.99-1.09)	1.06(1.02-1.11)	0.90(0.86-0.94)	-
P-value	0.143	<0.005	0.154	0.011	<0.005	-
**Smoking**						
Never	1.00	1.00	1.00	-	1.00	1.00
Ex-smokers	0.96(0.91-1.01)	0.96(0.91-1.01)	0.98(0.93-1.03)	-	0.87(0.82-0.92)	0.95(0.89-1.00)
Current	0.82(0.78-0.87)	0.86(0.81-0.91)	1.10(1.04-1.15)	-	0.85(0.79-0.90)	0.89(0.84-0.95)
P-value	<0.005	<0.005	<0.005	-	<0.005	<0.005
**Alcohol intake**						
Abstainer	1.00	1.00	1.00	-	1.00	1.00
Moderate	0.97(0.92-1.01)	0.96(0.92-1.01)	1.04(1.00-1.09)	-	1.14(1.09-1.20)	1.09(1.04-1.14)
Binge drinker	0.90(0.85-0.95)	0.93(0.88-0.99)	1.08(1.03-1.14)	-	1.10(1.04-1.17)	1.07(1.01-1.13)
P-value	<0.005	0.032	<0.005	-	<0.005	<0.005
**Self-Rated Health**						
Very good/Good	1.00	1.00	1.00	-	1.00	1.00
Fair	0.91(0.87-0.95)	0.93(0.89-0.97)	0.94(0.90-0.98)	-	0.83(0.79-0.87)	0.94(0.89-0.98)
Poor/Very poor	0.87(0.80-0.94)	0.91(0.84-0.99)	0.88(0.82-0.94)	-	0.72(0.66-0.78)	0.88(0.81-0.96)
P-value	<0.005	<0.005	<0.005	-	<0.005	<0.005
**Multimorbidity**						
0 or 1	1.00	-	1.00	1.00	1.00	-
2	1.05(0.99-1.11)	-	0.95(0.88-1.02)	0.93(0.88-0.98)	0.90(0.84-0.97)	-
3	1.13(1.05-1.22)	-	0.86(0.78-0.94)	0.96(0.89-1.04)	0.86(0.79-0.94)	-
4+	1.09(1.00-1.19)	-	0.86(0.78-0.94)	0.87(0.79-0.95)	0.83(0.75-0.91)	-
P-value	<0.005	-	<0.005	<0.005	<0.005	-

P-value to the Wald Test.

-: Variables not statistically significant in the model.

[a] Black(a), brown(a), indigenous.

[b] single, divorced, separated, widowed
