# Supplementary material for: Health, lifestyle and sociodemographic characteristics are associated with Brazilian dietary patterns: Brazilian National Health Survey
Source: PLoS One. 2021 Feb 16;16(2):e0247078. doi: 10.1371/journal.pone.0247078 (PMC7886222; doi:10.1371/journal.pone.0247078)
Supplement: S7 Table — Comparison between quartile 1 and quartile 2 for each dietary pattern. (PDF) [file pone.0247078.s007.pdf]

S7 Table. Associations between dietary patterns, lifestyle, health and sociodemographic characteristics in the Southeast Region of Brazil. Comparison between quartile 1 and quartile 2 for each dietary pattern.

DIETARY PATTERNS	HEALTHY		PROTEIN		WESTEN	
**Prevalence Ratio**	**Crude (95%CI)**	**Adjusted (95%CI)**	**Crude (95%CI)**	**Adjusted (95%CI)**	**Crude (95%CI)**	**Adjusted (95%CI)**
**Sample Size (n)**	**5,557**		**6,012**		**6,167**	
**Estimated Population Size (N)**	**25,954,162**		**24,938,817**		**26,028,704**	
**Age groups (years)**						
60+	1.00	1.00	1.00	1.00	1.00	1.00
18-24	0.80(0.70-0.91)	0.72(0.62-0.83)	1,08(0,96-1,21)	1,18(1,04-1,33)	1.23(1.06-1.42)	1.10(0.95-1.28)
25-39	0.92(0.84-1.00)	0.84(0.76-0.93)	1,09(1,01-1,19)	1,16(1,06-1,27)	1.27(1.15-1.41)	1.16(1.04-1.30)
40-59	0.91(0.84-0.99)	0.87(0.80-0.95)	1,05(0,96-1,15)	1,08(0,99-1,18)	1.06(0.95-1.17)	1.00(0.90-1.11)
P-value	0.009	<0.005	0.187	0.006	<0.005	<0.005
**Sex**						
Male	1.00	-	1.00	1.00	1.00	-
Female	1.07(1.00-1.14)	-	0,87(0,82-0,93)	0,88(0,82-0,94)	1.02(0.95-1.11)	-
P-value	0.041	-	<0.005	<0.005	0.531	-
**Skin Color/Race**						
White/Yellow	1.00	1.00	1.00	-	1.00	-
Others[a]	0.88(0.82-0.95)	0.91(0.84-0.97)	1,05(0,98-1,12)	-	0.98(0.91-1.06)	-
P-value	<0.005	0.006	0.144	-	0.614	-
**Marital status**						
Others[b]	1.00	-	1.00	1.00	1.00	-
Married	1.09(1.02-1.17)	-	1,09(1,02-1,16)	1,09(1,02-1,16)	1.05(0.98-1.14)	-
P-value	0.012	-	0.011	0.008	0.181	-
**Education**						
College	1.00	1.00	1.00	1.00	1.00	1.00
High School	0.89(0.82-0.98)	0.92(0.84-1.01)	1,23(1,13-1,34)	1,22(1,12-1,33)	0.84(0.77-0.92)	0.84(0.77-0.92)
Elementary School	0.88(0.80-0.97)	0.87(0.79-0.96)	1,22(1,11-1,33)	1,25(1,13-1,37)	0.70(0.64-0.77)	0.74(0.67-0.81)
Illiterate	0.80(0.69-0.92)	0.75(0.64-0.87)	1,18(1,03-1,36)	1,24(1,08-1,43)	0.59(0.50-0.71)	0.64(0.53-0.77)
P-value	0.008	<0.005	<0.005	<0.005	<0.005	<0.005
**Area of residence**						
Urban area	1.00	-	1.00	-	1.00	1.00
Rural area	0.92(0.79-1.07)	-	1,11(1,01-1,23)	-	0.77(0.68-0.87)	0.82(0.72-0.93)
P-value	0.286	-	0.025	-	<0.005	<0.005
**Economic Status**						
A-B	1.00	-	1.00	-	1.00	-
C	0.92(0.84-1.00)	-	1,04(0,96-1,12)	-	0.93(0.85-1.02)	-
D-E	0.93(0.85-1.01)	-	1,03(0,96-1,12)	-	0.87(0.78-0.96)	-
P-value	0.117	-	0.586	-	0.018	-

**Physical Activity**						
Sufficient	1.00	1.00	1.00	1.00	1.00	-
Insufficient	0.95(0.86-1.04)	0.93(0.85-1.02)	1,13(1,04-1,23)	1,14(1,04-1,23)	0.97(0.87-1.08)	-
None	0.93(0.85-1.00)	0.89(0.83-0.97)	1,09(1,02-1,18)	1,09(1,02-1,18)	0.94(0.87-1.02)	-
P-value	0.141	0.016	0.008	<0.005	0.348	-
**Smoking**						
Never	1.00	1.00	1.00	1.00	1.00	-
Ex-smokers	1.02(0.93-1.12)	0.98(0.89-1.07)	1,00(0,91-1,10)	0.98(0.89-1.07)	0.90(0.8-1.01)	-
Current	0.85(0.76-0.93)	0.85(0.77-0.94)	1,12(1,03-1,22)	0.85(0.77-0.94)	0.90(0.8-1.01)	-
P-value	<0.005	<0.005	0.034	<0.005	0.082	-
**Alcohol intake**						
Abstainer	1.00	-	1.00	-	1.00	-
Moderate	0.96(0.89-1.04)	-	1,01(0,94-1,09)	-	1.08(0.99-1.18)	-
Binge drinker	0.88(0.80-0.97)	-	1,09(0,99-1,19)	-	1.11(0.99-1.23)	-
P-value	0.029	-	0.204	-	0.068	-
**Self-Rated Health**						
Very good/Good	1.00	-	1.00	-	1.00	-
Fair	0.95(0.88-1.03)	-	1,04(0,96-1,11)	-	0.84(0.77-0.92)	-
Poor/Very poor	0.89(0.75-1.07)	-	1,00(0,87-1,14)	-	0.73(0.59-0.89)	-
P-value	0.250	-	0.639	-	<0.005	-
**Multimorbidity**						
0 or 1	1.00	-	1.00	-	1.00	-
2	1.05(0.96-1.15)	-	0,90(0,82-0,99)	-	0.98(0.88-1.08)	-
3	1.08(0.96-1.21)	-	0,94(0,83-1,07)	-	0.91(0.79-1.06)	-
4+	0.99(0.85-1.17)	-	0,91(0,80-1,04)	-	0.81(0.69-0.96)	-
P-value	0.499	-	0.107	-	0.068	-

P-value to the Wald Test.

-: Variables not statistically significant in the model.

[a] Black(a), brown(a), indigenous.

[b] single, divorced, separated, widowed
