# Supplementary material for: Health, lifestyle and sociodemographic characteristics are associated with Brazilian dietary patterns: Brazilian National Health Survey
Source: PLoS One. 2021 Feb 16;16(2):e0247078. doi: 10.1371/journal.pone.0247078 (PMC7886222; doi:10.1371/journal.pone.0247078)
Supplement: S8 Table — Comparison between quartile 1 and quartile 2 for each dietary pattern. (PDF) [file pone.0247078.s008.pdf]

S8 Table. Associations between dietary patterns, lifestyle, health and sociodemographic characteristics in the South Region of Brazil. Comparison between quartile 1 and quartile 2 for each dietary pattern.

DIETARY PATTERNS	HEALTHY		PROTEIN		WESTEN	
**Prevalence Ratio**	**Crude (95%CI)**	**Adjusted (95%CI)**	**Crude (95%CI)**	**Adjusted (95%CI)**	**Crude (95%CI)**	**Adjusted (95%CI)**
**Sample Size (n)**	**3,178**		**3,430**		**2,581**	
**Estimated Population Size (N)**	**9,608,729**		**8,666,567**		**7,741,367**	
**Age groups (years)**						
60+	1.00	-	1.00	-	1.00	1.00
18-24	0.79(0.66-0.94)	-	1.17(1.01-1.35)	-	1.49(1.29-1.72)	1.41(1.21-1.66)
25-39	0.93(0.80-1.08)	-	1.15(1.02-1.30)	-	1.25(1.09-1.44)	1.15(0.99-1.34)
40-59	0.99(0.87-1.13)	-	1.14(1.01-1.29)	-	1.15(1.02-1.29)	1.10(0.97-1.25)
P-value	0.031	-	0.092	-	<0.005	<0.005
**Sex**						
Male	1.00	1.00	1.00	1.00	1.00	-
Female	1.19(1.09-1.30)	1.13(1.03-1.25)	0.85(0.78-0.92)	0.87(0.80-0.95)	0.99(0.89-1.09)	-
P-value	<0.005	0.011	<0.005	<0.005	0.784	-
**Skin Color/Race**						
White/Yellow	1.00	1.00	1.00	1.00	1.00	-
Others[a]	0.83(0.74-0.93)	0.83(0.74-0.93)	1.10(0.98-1.23)	1.12(1.00-1.25)	0.83(0.73-0.94)	-
P-value	<0.005	<0.005	0.105	0.045	<0.005	-
**Marital status**						
Others[b]	1.00	1.00	1.00	1.00	1.00	-
Married	1.14(1.03-1.26)	1.13(1.02-1.24)	1.11(1.02-1.22)	1.10(1.01-1.21)	1.02(0.93-1.13)	-
P-value	0.009	0.015	0.021	0.030	0.639	-
**Education**						
College	1.00	-	1.00	-	1.00	1.00
High School	0.96(0.84-1.10)	-	1.06(0.94-1.20)	-	1.02(0.91-1.14)	1.03(0.93-1.15)
Elementary School	0.92(0.80-1.05)	-	1.08(0.96-1.21)	-	0.77(0.68-0.87)	0.84(0.74-0.95)
Illiterate	0.93(0.74-1.15)	-	0.98(0.79-1.23)	-	0.65(0.52-0.81)	0.76(0.60-0.96)
P-value	0.651	-	0.501	-	<0.005	<0.005
**Area of residence**						
Urban area	1.00	-	1.00	1.00	1.00	-
Rural area	1.10(0.99-1.23)	-	1.16(1.02-1.31)	1.14(1.01-1.29)	0.88(0.76-1.03)	-
P-value	0.083	-	0.018	0.034	0.107	-
**Economic Status**						
A-B	1.00	1.00	1.00	-	1.00	-
C	0.86(0.78-0.96)	0.87(0.79-0.96)	1.01(0.92-1.11)	-	0.97(0.86-1.10)	-
D-E	0.87(0.77-0.97)	0.86(0.77-0.96)	1.01(0.90-1.13)	-	0.86(0.76-0.97)	-
P-value	0.008	0.009	0.958	-	0.034	-

**Physical Activity**						
Sufficient	1.00	-	1.00	-	1.00	-
Insufficient	1.03(0.91-1.16)	-	1.07(0.95-1.21)	-	0.99(0.87-1.12)	-
None	1.04(0.94-1.15)	-	1.11(1.00-1.24)	-	0.94(0.84-1.06)	-
P-value	0.748	-	0.108	-	0.580	-
**Smoking**						
Never	1.00	-	1.00	-	1.00	1.00
Ex-smokers	0.94(0.83-1.08)	-	0.96(0.85-1.08)	-	0.94(0.85-1.04)	1.01(0.91-1.12)
Current	0.82(0.72-0.93)	-	1.13(1.01-1.26)	-	0.73(0.63-0.83)	0.78(0.68-0.89)
P-value	0.011	-	0.051	-	<0.005	<0.005
**Alcohol intake**						
Abstainer	1.00	1.00	1.00	-	1.00	-
Moderate	0.89(0.8-0.98)	0.90(0.81-0.99)	0.96(0.88-1.05)	-	1.12(1.02-1.23)	-
Binge drinker	0.72(0.6-0.86)	0.76(0.63-0.92)	0.96(0.82-1.13)	-	0.97(0.77-1.21)	-
P-value	<0.005	<0.005	0.627	-	0.046	-
**Self-Rated Health**						
Very good/Good	1.00	-	1.00	-	1.00	-
Fair	1.00(0.90-1.12)	-	0.96(0.87-1.07)	-	0.77(0.68-0.87)	-
Poor/Very poor	1.08(0.91-1.30)	-	0.98(0.83-1.15)	-	0.82(0.68-0.99)	-
P-value	0.660	-	0.771	-	<0.005	-
**Multimorbidity**						
0 or 1	1.00	-	1.00	1.00	1.00	-
2	1.03(0.90-1.17)	-	0.89(0.79-1.00)	0.89(0.78-1.00)	0.88(0.76-1.02)	-
3	1.22(1.04-1.42)	-	0.93(0.80-1.08)	0.94(0.81-1.10)	0.83(0.71-0.98)	-
4+	1.05(0.86-1.29)	-	0.77(0.64-0.93)	0.79(0.65-0.95)	0.86(0.73-1.03)	-
P-value	0.105	-	0.013	0.033	0.029	-

P-value to the Wald Test.

-: Variables not statistically significant in the model.

[a] Black(a), brown(a), indigenous.

[b] single, divorced, separated, widowed
