# Supplementary material for: Health, lifestyle and sociodemographic characteristics are associated with Brazilian dietary patterns: Brazilian National Health Survey
Source: PLoS One. 2021 Feb 16;16(2):e0247078. doi: 10.1371/journal.pone.0247078 (PMC7886222; doi:10.1371/journal.pone.0247078)
Supplement: S9 Table — Comparison between quartile 1 and quartile 2 for each dietary pattern. (PDF) [file pone.0247078.s009.pdf]

S9 Table. Associations between dietary patterns, lifestyle, health and sociodemographic characteristics in the Midwest Region of Brazil. Comparison between quartile 1 and quartile 2 for each dietary pattern.

**DIETARY PATTERNS**	HEALTHY		PROTEIN		WESTEN	
**Prevalence Ratio**	**Crude (95%CI)**	**Adjusted (95%CI)**	**Crude (95%CI)**	**Adjusted (95%CI)**	**Crude (95%CI)**	**Adjusted (95%CI)**
**Sample Size (n)**	**3,071**		**2,089**		**3,350**	
**Estimated Population Size (N)**	**45,011,918**		**2,696,589**		**4,665,108**	
**Age groups (years)**						
60+	1.00	-	1.00	-	1.00	1.00
18-24	0.91(0.79-1.05)	-	1.09(0.94-1.26)	-	1.59(1.39-1.82)	1.36(1.17-1.58)
25-39	0.92(0.82-1.04)	-	1.02(0.91-1.16)	-	1.28(1.13-1.45)	1.16(1.01-1.33)
40-59	0.94(0.83-1.06)	-	0.98(0.88-1.09)	-	1.15(1.02-1.30)	1.10(0.97-1.26)
P-value	0.512	-	0.498	-	<0.005	<0.005
**Sex**						
Male	1.00	1.00	1.00	-	1.00	-
Female	1.17(1.08-1.28)	1.17(1.07-1.27)	0.91(0.83-1.00)	-	0.99(0.92-1.07)	-
P-value	<0.005	<0.005	0.057	-	0.839	-
**Skin Color/Race**						
White/Yellow	1.00	1.00	1.00	1.00	1.00	-
Others[a]	0.90(0.82-0.99)	0.90(0.82-1.00)	1.12(1.02-1.22)	1.12(1.03-1.23)	1.02(0.93-1.11)	-
P-value	0.035	0.041	0.012	0.008	0.703	-
**Marital status**						
Others[b]	1.00	-	1.00	1.00	1.00	-
Married	1.00(0.92-1.09)	-	1.12(1.02-1.22)	1.12(1.03-1.23)	0.98(0.90-1.06)	-
P-value	0.968	-	0.018	0.012	0.625	-
**Education**						
College	1.00	-	1.00	-	1.00	1.00
High School	0.94(0.83-1.06)	-	1.07(0.94-1.21)	-	1.11(1.01-1.23)	1.11(1.01-1.23)
Elementary School	0.82(0.73-0.93)	-	1.17(1.04-1.32)	-	0.82(0.74-0.92)	0.93(0.83-1.05)
Illiterate	0.87(0.73-1.03)	-	1.13(0.96-1.32)	-	0.80(0.68-0.95)	0.98(0.82-1.17)
P-value	0.009	-	0.057	-	<0.005	<0.005
**Area of residence**						
Urban area	1.00	-	1.00	-	1.00	1.00
Rural area	0.99(0.90-1.09)	-	1.12(1.01-1.26)	-	0.78(0.68-0.88)	0.80(0.71-0.92)
P-value	0.823	-	0.038	-	<0.005	<0.005
**Economic Status**						
A-B	1.00	1.00	1.00	-	1.00	-
C	0.85(0.76-0.95)	0.85(0.76-0.95)	1.04(0.93-1.16)	-	0.94(0.85-1.05)	-
D-E	0.87(0.78-0.97)	0.87(0.78-0.97)	1.11(0.99-1.25)	-	0.90(0.81-1.00)	-
P-value	0.009	0.008	0.175	-	<0.005	-

**Physical Activity**						
Sufficient	1.00	-	1.00	-	1.00	-
Insufficient	1.06(0.95-1.19)	-	1.05(0.93-1.18)	-	1.04(0.93-1.16)	-
None	1.08(0.99-1.19)	-	1.05(0.96-1.16)	-	0.94(0.85-1.04)	-
P-value	0.219	-	0.465	-	0.251	-
**Smoking**						
Never	1.00	1.00	1.00	-	1.00	1.00
Ex-smokers	0.92(0.82-1.03)	0.94(0.84-1.06)	1.04(0.93-1.17)	-	0.76(0.67-0.87)	0.85(0.75-0.96)
Current	0.83(0.74-0.94)	0.86(0.76-0.97)	1.06(0.93-1.2)	-	0.79(0.67-0.87)	0.79(0.68-0.91)
P-value	0.006	0.034	0.594	-	<0.005	<0.005
**Alcohol intake**						
Abstainer	1.00	-	1.00	-	1.00	1.00
Moderate	1.03(0.94-1.13)	-	0.96(0.86-1.08)	-	1.01(0.91-1.12)	1.00(0.91-1.11)
Binge drinker	0.88(0.79-0.99)	-	0.96(0.83-1.11)	-	1.19(1.06-1.33)	1.18(1.06-1.33)
P-value	0.045	-	0.728	-	0.009	0.010
**Self-Rated Health**						
Very good/Good	1.00	-	1.00	-	1.00	-
Fair	0.98(0.89-1.09)	-	1.06(0.96-1.17)	-	0.87(0.80-0.96)	-
Poor/Very poor	0.98(0.83-1.16)	-	0.93(0.76-1.15)	-	0.71(0.58-0.87)	-
P-value	0.934	-	0.357	-	<0.005	-
**Multimorbidity**						
0 or 1	1.00	-	1.00	-	1.00	-
2	0.99(0.86-1.14)	-	1.00(0.88-1.14)	-	0.82(0.71-0.94)	-
3	1.00(0.82-1.23)	-	1.06(0.90-1.24)	-	0.72(0.58-0.89)	-
4+	1.08(0.90-1.30)	-	0.81(0.65-1.01)	-	0.81(0.67-0.99)	-
P-value	0.851	-	0.230	-	<0.005	-

P-value to the Wald Test.

-: Variables not statistically significant in the model.

[a] Black(a), brown(a), indigenous.

[b] single, divorced, separated, widowed
