# Supplementary material for: Health, lifestyle and sociodemographic characteristics are associated with Brazilian dietary patterns: Brazilian National Health Survey
Source: PLoS One. 2021 Feb 16;16(2):e0247078. doi: 10.1371/journal.pone.0247078 (PMC7886222; doi:10.1371/journal.pone.0247078)
Supplement: S10 Table — Comparison between quartile 1 and quartile 2 for each dietary pattern. (PDF) [file pone.0247078.s010.pdf]

S10 Table. Associations between dietary patterns, lifestyle, health and sociodemographic characteristics in the Northeast Region of Brazil. Comparison between quartile 1 and quartile 2 for each dietary pattern.

DIETARY PATTERNS	HEALTHY		PROTEIN		WESTEN	
**Prevalence Ratio**	**Crude (95%CI)**	**Adjusted (95%CI)**	**Crude (95%CI)**	**Adjusted (95%CI)**	**Crude (95%CI)**	**Adjusted (95%CI)**
**Sample Size (n)**	**10.196**		**11,113**		**11,155**	
**Estimated Population Size (N)**	**21,841,587**		**21,407,520**		**23,565,554**	
**Age groups (years)**						
60+	1.00	1.00	1.00	1.00	1.00	1.00
18-24	0.91(0.81-1.03)	0.67(0.58-0.76)	1.29(1.16-1.44)	1.27(1.13-1.42)	1.86(1.66-2.08)	1.60(1.41-1.80)
25-39	1.03(0.94-1.13)	0.80(0.73-0.88)	1.22(1.11-1.33)	1.21(1.10-1.33)	1.50(1.35-1.65)	1.31(1.17-1.45)
40-59	0.98(0.89-1.08)	0.87(0.79-0.96)	1.18(1.07-1.30)	1.17(1.06-1.29)	1.25(1.13-1.40)	1.14(1.02-1.27)
P-value	0.228	<0.005	<0.005	<0.005	<0.005	<0.005
**Sex**						
Male	1.00	1.00	1.00	1.00	1.00	-
Female	1.09(1.03-1.16)	1.06(1-1.13)	0.79(0.75-0.84)	0.80(0.76-0.85)	1.00(0.93-1.06)	-
P-value	<0.005	0.047	<0.005	<0.005	0.885	-
**Skin Color/Race**						
White/Yellow	1.00	-	1.00	-	1.00	-
Others[a]	0.92(0.86-0.99)	-	1.00(0.94-1.07)	-	1.01(0.93-1.10)	-
P-value	<0.005	-	0.935	-	0.746	-
**Marital status**						
Others[b]	1.00	-	1.00	-	1.00	-
Married	1.05(0.98-1.12)	-	1.04(0.97-1.11)	-	0.93(0.87-0.99)	-
P-value	0.190	-	0.273	-	0.034	-
**Education**						
College	1.00	1.00	1.00	1.00	1.00	1.00
High School	0.94(0.86-1.04)	0.98(0.9-1.07)	1.18(1.09-1.29)	1.17(1.08-1.28)	1.00(0.91-1.09)	0.99(0.90-1.08)
Elementary School	0.80(0.73-0.89)	0.85(0.77-0.94)	1.15(1.06-1.26)	1.21(1.11-1.32)	0.79(0.72-0.87)	0.88(0.81-0.97)
Illiterate	0.67(0.59-0.76)	0.68(0.59-0.78)	1.07(0.95-1.20)	1.21(1.07-1.36)	0.64(0.56-0.73)	0.80(0.69-0.92)
P-value	<0.005	<0.005	<0.005	<0.005	<0.005	<0.005
**Area of residence**						
Urban area	1.00	1.00	1.00	-	1.00	1.00
Rural area	0.76(0.70-0.83)	0.81(0.74-0.89)	1.08(1-1.17)	-	0.83(0.75-0.91)	0.86(0.78-0.95)
P-value	<0.005	<0.005	0.050	-	<0.005	<0.005
**Economic Status**						
A-B	1.00	-	1.00	-	1.00	-
C	1.02(0.87-1.20)	-	1.05(0.95-1.15)	-	0.87(0.77-0.97)	-
D-E	0.86(0.73-1.01)	-	1.03(0.94-1.13)	-	0.79(0.71-0.88)	-
P-value	<0.005	-	0.616	-	<0.005	-

**Physical Activity**						
Sufficient	1.00	-	1.00	-	1.00	1.00
Insufficient	1.03(0.94-1.12)	-	0.95(0.88-1.03)	-	0.87(0.80-0.96)	0.92(0.84-1.00)
None	0.94(0.87-1.01)	-	0.96(0.88-1.04)	-	0.81(0.75-0.88)	0.90(0.84-0.97)
P-value	0.207	-	0.415	-	<0.005	0.010
**Smoking**						
Never	1.00	1.00	1.00	-	1.00	-
Ex-smokers	0.91(0.84-1.00)	0.92(0.84-1.01)	0.97(0.90-1.05)	-	0.80(0.74-0.87)	-
Current	0.74(0.67-0.83)	0.78(0.70-0.87)	1.09(1.00-1.20)	-	0.84(0.76-0.93)	-
P-value	<0.005	<0.005	0.098	-	<0.005	-
**Alcohol intake**						
Abstainer	1.00	-	1.00	-	1.00	1.00
Moderate	0.95(0.88-1.03)	-	1.08(1.01-1.16)	-	1.25(1.15-1.35)	1.16(1.07-1.25)
Binge drinker	0.97(0.88-1.06)	-	1.18(1.09-1.27)	-	1.15(1.05-1.26)	1.06(0.97-1.15)
P-value	0.416	-	<0.005	-	<0.005	<0.005
**Self-Rated Health**						
Very good/Good	1.00	1.00	1.00	1.00	1.00	-
Fair	0.89(0.83-0.95)	0.89(0.82-0.96)	0.90(0.84-0.97)	0.93(0.87-0.99)	0.85(0.79-0.92)	-
Poor/Very poor	0.84(0.75-0.95)	0.88(0.78-0.99)	0.82(0.74-0.91)	0.86(0.77-0.95)	0.72(0.64-0.81)	-
P-value	<0.005	<0.005	<0.005	0.006	<0.005	-
**Multimorbidity**						
0 or 1	1.00	-	1.00	-	1.00	-
2	1.04(0.94-1.15)	-	0.84(0.77-0.93)	-	0.80(0.69-0.93)	-
3	1.09(0.95-1.25)	-	0.86(0.75-0.98)	-	0.77(0.65-0.92)	-
4+	1.13(0.98-1.30)	-	0.72(0.61-0.85)	-	0.75(0.62-0.91)	-
P-value	0.204	-	<0.005	-	<0.005	-

P-value to the Wald Test.

-: Variables not statistically significant in the model.

[a] Black(a), brown(a), indigenous.

[b] single, divorced, separated, widowed
