# Supplementary material for: Health, lifestyle and sociodemographic characteristics are associated with Brazilian dietary patterns: Brazilian National Health Survey
Source: PLoS One. 2021 Feb 16;16(2):e0247078. doi: 10.1371/journal.pone.0247078 (PMC7886222; doi:10.1371/journal.pone.0247078)
Supplement: S11 Table — Comparison between quartile 1 and quartile 2 for each dietary pattern. (PDF) [file pone.0247078.s011.pdf]

S11 Table. Associations between dietary patterns, lifestyle, health and sociodemographic characteristics in the North Region of Brazil. Comparison between quartile 1 and quartile 2 for each dietary pattern.

DIETARY PATTERNS	HEALTHY		PROTEIN		WESTEN	
**Prevalence Ratio**	**Crude (95%CI)**	**Adjusted (95%CI)**	**Crude (95%CI)**	**Adjusted (95%CI)**	**Crude (95%CI)**	**Adjusted (95%CI)**
**Sample Size (n)**	**8,099**		**7,457**		**6,648**	
**Estimated Population Size (N)**	**7,187,569**		**6,850,478**		**6,238,495**	
**Age groups (years)**						
60+	1.00	1.00	1.00	-	1.00	1.00
18-24	0.87(0.72-1.05)	0.70(0.57-0.86)	1.19(0.98-1.45)	-	1.46(1.24-1.72)	1.46(1.24-1.72)
25-39	1.00(0.85-1.17)	0.83(0.70-1.00)	1.34(1.09-1.64)	-	1.32(1.15-1.52)	1.33(1.16-1.53)
40-59	1.13(0.96-1.32)	1.03(0.87-1.21)	1.13(0.91-1.41)	-	1.01(0.87-1.18)	1.01(0.87-1.18)
P-value	0.007	<0.005	0.009	-	<0.005	<0.005
**Sex**						
Male	1.00	-	1.00	-	1.00	-
Female	1.11(1.01-1.22)	-	1.01(0.91-1.13)	-	1.00(0.91-1.10)	-
P-value	0.033	-	0.818	-	0.994	-
**Skin Color/Race**						
White/Yellow	1.00	-	1.00	1.00	1.00	-
Others[a]	0.84(0.75-0.93)	-	0.74(0.65-0.83)	0.78(0.70-0.87)	0.97(0.87-1.08)	-
P-value	<0.005	-	<0.005	<0.005	0.597	-
**Marital status**						
Others[b]	1.00	-	1.00	-	1.00	-
Married	1.02(0.92-1.13)	-	1.04(0.92-1.16)	-	0.94(0.86-1.03)	-
P-value	0.727	-	0.541	-	0.216	-
**Education**						
College	1.00	1.00	1.00	-	1.00	-
High School	0.85(0.75-0.96)	0.90(0.80-1.01)	1.05(0.90-1.21)	-	1.01(0.89-1.15)	-
Elementary School	0.68(0.59-0.77)	0.70(0.61-0.80)	0.80(0.68-0.95)	-	0.78(0.68-0.90)	-
Illiterate	0.65(0.53-0.81)	0.63(0.50-0.80)	0.77(0.60-0.98)	-	0.73(0.62-0.87)	-
P-value	<0.005	<0.005	<0.005	-	<0.005	-
**Area of residence**						
Urban area	1.00	1.00	1.00	1.00	1.00	1.00
Rural area	0.64(0.54-0.76)	0.69(0.58-0.83)	0.59(0.47-0.75)	0.60(0.48-0.76)	0.69(0.59-0.80)	0.68(0.58-0.80)
P-value	<0.005	<0.005	<0.005	<0.005	<0.005	<0.005
**Economic Status**						
A-B	1.00	-	1.00	-	1.00	-
C	0.87(0.77-0.99)	-	0.90(0.79-1.03)	-	0.96(0.86-1.07)	-
D-E	0.75(0.65-0.86)	-	0.76(0.65-0.88)	-	0.84(0.75-0.94)	-
P-value	<0.005	-	<0.005	-	<0.005	-

**Physical Activity**						
Sufficient	1.00	-	1.00	-	1.00	-
Insufficient	1.00(0.87-1.15)	-	0.94(0.81-1.09)	-	0.94(0.82-1.08)	-
None	0.90(0.80-1.01)	-	0.91(0.79-1.06)	-	0.82(0.74-0.91)	-
P-value	0.140	-	0.439	-	<0.005	-
**Smoking**						
Never	1.00	-	1.00	-	1.00	-
Ex-smokers	0.89(0.77-1.02)	-	0.93(0.80-1.08)	-	0.90(0.8-1.02)	-
Current	0.91(0.77-1.08)	-	0.92(0.78-1.10)	-	0.88(0.77-1.01)	-
P-value	0.172	-	0.457	-	0.016	-
**Alcohol intake**						
Abstainer	1.00	-	1.00	-	1.00	-
Moderate	0.96(0.85-1.09)	-	1.03(0.89-1.19)	-	1.09(0.99-1.20)	-
Binge drinker	1.07(0.93-1.23)	-	1.11(0.95-1.30)	-	1.16(1.00-1.35)	-
P-value	0.491	-	0.419	-	0.072	-
**Self-Rated Health**						
Very good/Good	1.00	-	1.00	-	1.00	-
Fair	0.91(0.81-1.02)	-	0.84(0.74-0.97)	-	0.86(0.78-0.94)	-
Poor/Very poor	0.80(0.65-0.99)	-	0.84(0.65-1.09)	-	0.72(0.59-0.88)	-
P-value	0.049	-	0.039	-	<0.005	-
**Multimorbidity**						
0 or 1	1.00	-	1.00	1.00	1.00	-
2	1.04(0.89-1.21)	-	0.99(0.84-1.17)	0.96(0.81-1.14)	0.87(0.74-1.02)	-
3	1.17(0.96-1.42)	-	0.79(0.62-1.00)	0.77(0.60-0.97)	0.73(0.58-0.92)	-
4+	1.39(1.03-1.89)	-	0.63(0.43-0.93)	0.59(0.40-0.87)	0.66(0.44-0.98)	-
P-value	0.100	-	0.028	0.010	0.006	-

P-value to the Wald Test.

-: Variables not statistically significant in the model.

[a] Black(a), brown(a), indigenous.

[b] single, divorced, separated, widowed
