# Supplementary material for: Health, lifestyle and sociodemographic characteristics are associated with Brazilian dietary patterns: Brazilian National Health Survey
Source: PLoS One. 2021 Feb 16;16(2):e0247078. doi: 10.1371/journal.pone.0247078 (PMC7886222; doi:10.1371/journal.pone.0247078)
Supplement: S12 Table — Comparison between quartile 1 and quartile 3 for each dietary pattern. (PDF) [file pone.0247078.s012.pdf]

S12 Table. Associations between dietary patterns, lifestyle, health and sociodemographic characteristics in Brazil. Comparison between quartile 1 and quartile 3 for each dietary pattern.

DIETARY PATTERNS	HEALTHY		PROTEIN		WESTEN	
**Prevalence Ratio**	**Crude (95%CI)**	**Adjusted (95%CI)**	**Crude (95%CI)**	**Adjusted (95%CI)**	**Crude (95%CI)**	**Adjusted (95%CI)**
**Sample Size (n)**	**30,102**		**30,102**		**30,102**	
**Estimated Population Size (N)**	**70,971,387**		**68,774,896**		**69,681,666**	
**Age groups (years)**						
60+	1.00	1.00	1.00	1.00	1.00	1.00
18-24	0.70(0.65-0.76)	0.60(0.56-0.65)	1.32(1.24-1.40)	1.41(1.31-1.50)	1.83(1.70-1.96)	1.55(1.43-1.67)
25-39	0.83(0.79-0.87)	0.71(0.67-0.75)	1.28(1.22-1.36)	1.34(1.27-1.42)	1.58(1.49-1.68)	1.37(1.28-1.46)
40-59	0.93(0.88-0.97)	0.84(0.80-0.88)	1.19(1.13-1.26)	1.19(1.13-1.26)	1.22(1.14-1.31)	1.12(1.04-1.19)
P-value	<0.005	<0.005	<0.005	<0.005	<0.005	<0.005
**Sex**						
Male	1.00	1.00	1.00	1.00	1.00	-
Female	1.20(1.15-1.25)	1.14(1.10-1.18)	0.80(0.77-0.82)	0.82(0.79-0.85)	0.94(0.91-0.98)	-
P-value	<0.005	<0.005	<0.005	<0.005	0.005	-
**Skin Color/Race**						
White/Yellow	1.00	1.00	1.00	-	1.00	1.00
Others[a]	0.79(0.76-0.82)	0.94(0.90-0.97)	0.94(0.91-0.97)	-	0.83(0.80-0.86)	0.94(0.90-0.98)
P-value	<0.005	<0.005	0.8816	-	<0.005	<0.005
**Marital status**						
Others[b]	1.00	1.00	1.00	1.00	1.00	-
Married	1.11(1.06-1.15)	1.09(1.05-1.13)	1.06(1.02-1.09)	1.06(1.03-1.10)	0.96(0.92-1.00)	-
P-value	<0.005	<0.005	<0.005	<0.005	0.050	-
**Education**						
College	1.00	1.00	1.00	1.00	1.00	1.00
High School	0.84(0.80-0.88)	0.91(0.87-0.95)	1.22(1.16-1.29)	1.24(1.18-1.31)	0.91(0.87-0.95)	0.94(0.90-0.98)
Elementary School	0.73(0.70-0.77)	0.77(0.73-0.82)	1.22(1.16-1.29)	1.35(1.28-1.42)	0.67(0.63-0.70)	0.81(0.77-0.85)
Illiterate	0.63(0.58-0.69)	0.67(0.61-0.73)	1.03(0.95-1.11)	1.34(1.24-1.45)	0.45(0.41-0.49)	0.67(0.61-0.75)
P-value	<0.005	<0.005	<0.005	<0.005	<0.005	<0.005
**Area of residence**						
Urban area	1.00	1.00	1.00	-	1.00	1.00
Rural area	0.70(0.66-0.75)	0.86(0.81-0.92)	0.98(0.93-1.04)	-	0.66(0.61-0.71)	0.78(0.73-0.84)
P-value	<0.005	<0.005	0.530	-	<0.005	<0.005
**Economic Status**						
A-B	1.00	1.00	1.00	-	1.00	-
C	0.86(0.82-0.90)	0.97(0.92-1.02)	1.01(0.97-1.06)	-	0.85(0.81-0.89)	-
D-E	0.73(0.69-0.77)	0.92(0.87-0.97)	0.92(0.87-0.96)	-	0.74(0.70-0.78)	-
P-value	<0.005	0.01	<0.005	-	<0.005	-

**Physical Activity**						
Sufficient	1.00	1.00	1.00	-	1.00	-
Insufficient	0.95(0.90-1.00)	0.91(0.86-0.95)	1.03(0.99-1.08)	-	0.97(0.92-1.02)	-
None	0.91(0.87-0.95)	0.87(0.83-0.91)	1.01(0.97-1.05)	-	0.87(0.83-0.91)	-
P-value	<0.005	<0.005	0.335	-	<0.005	-
**Smoking**						
Never	1.00	1.00	1.00	1.00	1.00	-
Ex-smokers	0.94(0.89-0.99)	0.92(0.88-0.97)	0.99(0.95-1.04)	1.01(0.97-1.06)	0.84(0.80-0.89)	-
Current	0.77(0.72-0.82)	0.82(0.78-0.88)	1.17(1.13-1.22)	1.12(1.07-1.16)	0.89(0.84-0.95)	-
P-value	<0.005	<0.005	<0.005	<0.005	<0.005	-
**Alcohol intake**						
Abstainer	1.00	1.00	1.00	-	1.00	1.00
Moderate	0.98(0.94-1.03)	0.97(0.93-1.01)	1.11(1.07-1.16)	-	1.21(1.16-1.27)	1.08(1.04-1.13)
Binge drinker	0.82(0.77-0.87)	0.90(0.84-0.96)	1.18(1.12-1.24)	-	1.23(1.17-1.30)	1.11(1.05-1.16)
P-value	<0.005	0.01	<0.005	-	<0.005	<0.005
**Self-Rated Health**						
Very good/Good	1.00	1.00	1.00	-	1.00	1.00
Fair	0.87(0.83-0.91)	0.87(0.83-0.91)	0.92(0.88-0.95)	-	0.75(0.71-0.78)	0.92(0.88-0.96)
Poor/Very poor	0.78(0.72-0.85)	0.81(0.74-0.88)	0.82(0.76-0.89)	-	0.60(0.55-0.67)	0.83(0.76-0.92)
P-value	<0.005	<0.005	<0.005	-	<0.005	<0.005
**Multimorbidity**						
0 or 1	1.00	1.00	1.00	1.00	1.00	-
2	1.14(1.08-1.2)	1.08(1.02-1.14)	0.85(0.79-0.93)	0.92(0.86-0.97)	0.83(0.78-0.88)	-
3	1.24(1.16-1.32)	1.13(1.06-1.21)	0.81(0.74-0.88)	0.90(0.83-0.98)	0.78(0.71-0.85)	-
4+	1.24(1.15-1.32)	1.14(1.06-1.23)	0.81(0.74-0.88)	0.87(0.79-0.95)	0.76(0.68-0.84)	-
P-value	<0.005	<0.005	<0.005	<0.005	<0.005	-

P-value to the Wald Test.

-: Variables not statistically significant in the model.

[a] Black(a), brown(a), indigenous.

[b] single, divorced, separated, widowed
