# Supplementary material for: Health, lifestyle and sociodemographic characteristics are associated with Brazilian dietary patterns: Brazilian National Health Survey
Source: PLoS One. 2021 Feb 16;16(2):e0247078. doi: 10.1371/journal.pone.0247078 (PMC7886222; doi:10.1371/journal.pone.0247078)
Supplement: S13 Table — Comparison between quartile 1 and quartile 3 for each dietary pattern. (PDF) [file pone.0247078.s013.pdf]

S13 Table. Associations between dietary patterns, lifestyle, health and sociodemographic characteristics in the Southeast Region of Brazil. Comparison between quartile 1 and quartile 3 for each dietary pattern.

**DIETARY PATTERNS**	**HEALTHY**		**PROTEIN**		**WESTEN**	
**Prevalence Ratio**	**Crude (95%CI)**	**Adjusted (95%CI)**	**Crude (95%CI)**	**Adjusted (95%CI)**	**Crude (95%CI)**	**Adjusted (95%CI)**
**Sample Size (n)**	**6,167**		**6,644**		**6,688**	
**Estimated Population Size (N)**	**27,759,421**		**28,557,458**		**28,733,969**	
**Age groups (years)**						
60+	1.00	1.00	1.00	1.00	1.00	1.00
18-24	0.72(0.63-0.82)	0.63(0.55-0.71)	1.36(1.23-1.50)	1.49(1.34-1.65)	1.65(1.47-1.85)	1.34(1.18-1.53)
25-39	0.82(0.76-0.89)	0.70(0.64-0.76)	1.34(1.23-1.46)	1.45(1.33-1.59)	1.55(1.41-1.71)	1.30(1.17-1.44)
40-59	0.88(0.82-0.95)	0.80(0.74-0.86)	1.17(1.07-1.27)	1.20(1.10-1.31)	1.20(1.08-1.33)	1.08(0.97-1.20)
P-value	<0.005	<0.005	<0.005	<0.005	<0.005	<0.005
**Sex**						
Male	1.00	1.00	1.00	1.00	1.00	-
Female	1.16(1.09-1.24)	1.15(1.08-1.22)	0.85(0.81-0.90)	0.87(0.82-0.92)	0.92(0.86-0.99)	-
P-value	<0.005	<0.005	<0.005	<0.005	0.023	-
**Skin Color/Race**						
White/Yellow	1.00	-	1.00	-	1.00	-
Others[a]	0.89(0.83-0.94)	-	1.12(1.06-1.18)	-	0.93(0.86-0.99)	-
P-value	<0.005	-	<0.005	-	0.028	-
**Marital status**						
Others[b]	1.00	1.00	1.00	1.00	1.00	-
Married	1.13(1.06-1.21)	1.11(1.04-1.18)	1.05(0.99-1.11)	1.06(1.00-1.12)	1.00(0.94-1.07)	-
P-value	<0.005	<0.005	0.106	0.045	0.917	-
**Education**						
College	1.00	1.00	1.00	1.00	1.00	1.00
High School	0.87(0.81-0.94)	0.90(0.83-0.97)	1.33(1.23-1.43)	1.29(1.19-1.39)	0.87(0.81-0.94)	0.87(0.81-0.94)
Elementary School	0.84(0.77-0.91)	0.80(0.73-0.87)	1.35(1.24-1.47)	1.42(1.30-1.54)	0.66(0.61-0.72)	0.76(0.70-0.83)
Illiterate	0.77(0.66-0.90)	0.71(0.60-0.83)	1.20(1.05-1.37)	1.37(1.20-1.57)	0.46(0.38-0.56)	0.58(0.47-0.71)
P-value	<0.005	<0.005	<0.005	<0.005	<0.005	<0.005
**Area of residence**						
Urban area	1.00	-	1.00	1.00	1.00	1.00
Rural area	0.84(0.74-0.96)	-	1.24(1.15-1.33)	1.13(1.05-1.22)	0.75(0.64-0.87)	0.82(0.70-0.96)
P-value	0.007	-	<0.005	<0.005	<0.005	0.011

**Economic Status**						
A-B	1.00	-	1.00	-	1.00	-
C	0.91(0.84-0.98)	-	1.06(1.00-1.12)	-	0.92(0.85-0.99)	-
D-E	0.90(0.83-0.98)	-	1.03(0.96-1.10)	-	0.88(0.80-0.96)	-
P-value	0.025	-	0.195	-	0.015	-
**Physical Activity**						
Sufficient	1.00	1.00	1.00	1.00	1.00	-
Insufficient	0.93(0.85-1.02)	0.90(0.82-0.98)	0.93(0.85-1.02)	1.11(1.04-1.18)	0.93(0.85-1.02)	-
None	0.92(0.85-1.00)	0.89(0.82-0.96)	1.02(0.96-1.08)	1.05(0.99-1.12)	0.87(0.81-0.94)	-
P-value	0.086	<0.005	0.073	0.006	<0.005	-
**Smoking**						
Never	1.00	1.00	1.00	1.00	1.00	-
Ex-smokers	0.96(0.88-1.05)	0.94(0.86-1.02)	0.99(0.92-1.07)	1.01(0.93-1.09)	0.88(0.81-0.97)	-
Current	0.86(0.78-0.94)	0.90(0.82-0.99)	1.15(1.08-1.23)	1.11(1.04-1.18)	0.96(0.87-1.05)	-
P-value	0.006	0.056	<0.005	0.008	0.024	-
**Alcohol intake**						
Abstainer	1.00	-	1.00	-	1.00	1.00
Moderate	0.97(0.91-1.04)	-	1.05(0.98-1.12)	-	1.19(1.11-1.28)	1.10(1.02-1.18)
Binge drinker	0.86(0.78-0.96)	-	1.16(1.08-1.25)	-	1.28(1.18-1.39)	1.15(1.06-1.24)
P-value	0.018	-	<0.005	-	<0.005	<0.005
**Self-Rated Health**						
Very good/Good	1.00	1.00	1.00	-	1.00	1.00
Fair	0.95(0.89-1.02)	0.92(0.86-0.99)	0.99(0.93-1.05)	-	0.79(0.73-0.86)	0.94(0.86-1.02)
Poor/Very poor	0.88(0.76-1.01)	0.87(0.76-1.00)	0.95(0.84-1.08)	-	0.61(0.49-0.75)	0.79(0.64-0.96)
P-value	0.11	0.025	0.727	-	<0.005	0.043
**Multimorbidity**						
0 or 1	1.00	-	1.00	-	1.00	-
2	1.13(1.03-1.23)	-	0.85(0.78-0.93)	-	0.88(0.80-0.96)	-
3	1.16(1.04-1.29)	-	0.89(0.79-1.00)	-	0.74(0.64-0.87)	-
4+	1.15(1.04-1.28)	-	0.82(0.71-0.93)	-	0.70(0.58-0.84)	-
P-value	<0.005	-	<0.005	-	<0.005	-

P-value to the Wald Test.

-: Variables not statistically significant in the model.

[a] Black(a), brown(a), indigenous.

[b] single, divorced, separated, widowed
