# Supplementary material for: Health, lifestyle and sociodemographic characteristics are associated with Brazilian dietary patterns: Brazilian National Health Survey
Source: PLoS One. 2021 Feb 16;16(2):e0247078. doi: 10.1371/journal.pone.0247078 (PMC7886222; doi:10.1371/journal.pone.0247078)
Supplement: S14 Table — Comparison between quartile 1 and quartile 3 for each dietary pattern. (PDF) [file pone.0247078.s014.pdf]

S14 Table. Associations between dietary patterns, lifestyle, health and sociodemographic characteristics in the South Region of Brazil. Comparison between quartile 1 and quartile 3 for each dietary pattern.

DIETARY PATTERNS	HEALTHY		PROTEIN		WESTEN	
**Prevalence Ratio**	**Crude (95%CI)**	**Adjusted (95%CI)**	**Crude (95%CI)**	**Adjusted (95%CI)**	**Crude (95%CI)**	**Adjusted (95%CI)**
**Sample Size (n)**	**3,478**		**3,490**		**3,039**	
**Estimated Population Size (N)**	**10,374,128**		**9,690,513**		**8,673,456**	
**Age groups (years)**						
60+	1.00	1.00	1.00	1.00	1.00	1.00
18-24	0.62(0.52-0.75)	0.59(0.49-0.70)	1.20(1.04-1.38)	1.21(1.04-1.41)	1.55(1.36-1.77)	1.32(1.15-1.52)
25-39	0.80(0.71-0.90)	0.75(0.66-0.85)	1.17(1.05-1.30)	1.17(1.04-1.31)	1.38(1.23-1.55)	1.20(1.07-1.36)
40-59	0.95(0.85-1.06)	0.93(0.84-1.03)	1.19(1.07-1.33)	1.20(1.08-1.33)	1.17(1.04-1.31)	1.1(0.98-1.24)
P-value	<0.005	<0.005	0.011	0.008	<0.005	<0.005
**Sex**						
Male	1.00	1.00	1.00	1.00	1.00	-
Female	1.27(1.17-1.38)	1.19(1.09-1.29)	0.78(0.73-0.84)	0.81(0.75-0.87)	1.04(0.95-1.13)	-
P-value	<0.005	<0.005	<0.005	<0.005	0.453	-
**Skin Color/Race**						
White/Yellow	1.00	1.00	1.00	1.00	1.00	1.00
Others[a]	0.79(0.70-0.88)	0.88(0.78-0.99)	1.22(1.12-1.32)	1.16(1.07-1.27)	0.90(0.82-0.98)	0.91(0.83-0.99)
P-value	<0.005	0.036	<0.005	<0.005	0.018	0.029
**Marital status**						
Others[b]	1.00	-	1.00	1.00	1.00	-
Married	1.17(1.07-1.29)	-	1.14(1.06-1.24)	1.10(1.02-1.19)	1.00(0.92-1.08)	-
P-value	0.005	-	<0.005	0.012	0.963	-
**Education**						
College	1.00	1.00	1.00	1.00	1.00	1.00
High School	0.87(0.77-0.98)	0.91(0.81-1.02)	1.28(1.14-1.45)	1.25(1.11-1.39)	0.97(0.89-1.05)	0.97(0.89-1.05)
Elementary School	0.78(0.69-0.89)	0.73(0.64-0.83)	1.30(1.15-1.47)	1.31(1.17-1.47)	0.73(0.67-0.80)	0.84(0.76-0.92)
Illiterate	0.87(0.73-1.04)	0.72(0.59-0.87)	1.14(0.93-1.41)	1.23(0.99-1.53)	0.54(0.44-0.68)	0.66(0.53-0.83)
P-value	<0.005	<0.005	<0.005	<0.005	<0.005	<0.005
**Area of residence**						
Urban area	1.00	1.00	1.00	-	1.00	1.00
Rural area	1.10(1.00-1.21)	1.18(1.07-1.30)	1.12(1.01-1.25)	-	0.76(0.66-0.87)	0.81(0.7-0.94)
P-value	0.062	<0.005	0.033	-	<0.005	0.005
**Economic Status**						
A-B	1.00	1	1.00	-	1.00	-
C	0.82(0.74-0.92)	0.87(0.78-0.97)	1.11(1.01-1.22)	-	0.90(0.82-1.00)	-
D-E	0.87(0.77-0.97)	0.89(0.80-0.99)	1.08(0.96-1.20)	-	0.88(0.79-0.97)	-
P-value	<0.005	0.025	0.090	-	0.038	-

**Physical Activity**						
Sufficient	1.00	-	1.00	1.00	1.00	-
Insufficient	0.93(0.85-1.02)	-	0.93(0.85-1.02)	1.09(0.99-1.19)	0.93(0.85-1.02)	-
None	0.98(0.89-1.08)	-	1.08(0.99-1.17)	1.13(1.04-1.22)	0.9(0.82-0.99)	-
P-value	0.812	-	0.199	0.011	0.090	-
**Smoking**						
Never	1.00	1.00	1.00	1.00	1.00	-
Ex-smokers	1.00(0.89-1.11)	1.02(0.92-1.12)	1.04(0.95-1.14)	1.02(0.94-1.11)	0.92(0.85-1.00)	-
Current	0.68(0.58-0.79)	0.75(0.65-0.87)	1.23(1.13-1.33)	1.14(1.05-1.24)	0.81(0.72-0.91)	-
P-value	<0.005	<0.005	<0.005	0.01	<0.005	-
**Alcohol intake**						
Abstainer	1.00	1.00	1.00	-	1.00	-
Moderate	0.94(0.86-1.03)	0.99(0.91-1.09)	1.04(0.97-1.12)	-	1.11(1.03-1.19)	-
Binge drinker	0.59(0.48-0.72)	0.70(0.57-0.85)	1.07(0.94-1.22)	-	1.00(0.83-1.21)	-
P-value	<0.005	<0.005	0.427	-	0.011	-
**Self-Rated Health**						
Very good/Good	1.00	-	1.00	-	1.00	1.00
Fair	0.98(0.89-1.07)	-	0.97(0.88-1.07)	-	0.76(0.68-0.84)	0.87(0.79-0.96)
Poor/Very poor	0.96(0.82-1.13)	-	0.94(0.78-1.13)	-	0.77(0.64-0.93)	0.92(0.76-1.12)
P-value	0.837	-	0.701	-	<0.005	0.021
**Multimorbidity**						
0 or 1	1.00	-	1.00	1	1.00	-
2	1.10(0.98-1.23)	-	0.87(0.77-0.97)	0.89(0.79-1.01)	0.81(0.72-0.92)	-
3	1.30(1.16-1.45)	-	0.84(0.71-0.99)	0.87(0.74-1.03)	0.81(0.69-0.94)	-
4+	1.14(0.97-1.33)	-	0.79(0.66-0.95)	0.82(0.69-0.99)	0.84(0.72-0.99)	-
P-value	<0.005	-	<0.005	0.053	<0.005	-

P-value to the Wald Test.

-: Variables not statistically significant in the model.

[a] Black(a), brown(a), indigenous.

[b] single, divorced, separated, widowed
