# Supplementary material for: Health, lifestyle and sociodemographic characteristics are associated with Brazilian dietary patterns: Brazilian National Health Survey
Source: PLoS One. 2021 Feb 16;16(2):e0247078. doi: 10.1371/journal.pone.0247078 (PMC7886222; doi:10.1371/journal.pone.0247078)
Supplement: S15 Table — Comparison between quartile 1 and quartile 3 for each dietary pattern. (PDF) [file pone.0247078.s015.pdf]

S15 Table. Associations between dietary patterns, lifestyle, health and sociodemographic characteristics in the Midwest Region of Brazil. Comparison between quartile 1 and quartile 3 for each dietary pattern.

**DIETARY PATTERNS**	**HEALTHY**		**PROTEIN**		**WESTEN**	
**Prevalence Ratio**	**Crude (95%CI)**	**Adjusted (95%CI)**	**Crude (95%CI)**	**Adjusted (95%CI)**	**Crude (95%CI)**	**Adjusted (95%CI)**
**Sample Size (n)**	**3,283**		**2,796**		**3,564**	
**Estimated Population Size (N)**	**4,924,729**		**3,766,131**		**5,072,427**	
**Age groups (years)**						
60+	1.00	1.00	1.00	1.00	1.00	1.00
18-24	0.81(0.71-0.93)	0.64(0.55-0.74)	1.28(1.16-1.41)	1.45(1.29-1.62)	2.01(1.76-2.30)	1.77(1.53-2.04)
25-39	0.90(0.81-1.01)	0.74(0.65-0.84)	1.14(1.04-1.25)	1.25(1.13-1.38)	1.66(1.47-1.87)	1.48(1.30-1.68)
40-59	0.97(0.88-1.07)	0.87(0.78-0.97)	1.11(1.00-1.22)	1.16(1.05-1.28)	1.28(1.13-1.45)	1.21(1.06-1.38)
P-value	0.010	<0.005	<0.005	<0.005	<0.005	<0.005
**Sex**						
Male	1.00	1.00	1.00	1.00	1.00	-
Female	1.28(1.18-1.39)	1.24(1.15-1.34)	0.89(0.84-0.95)	0.90(0.85-0.96)	0.93(0.86-1.00)	-
P-value	<0.005	<0.005	<0.005	<0.005	0.041	-
**Skin Color/Race**						
White/Yellow	1.00	-	1.00	-	1.00	-
Others[a]	0.88(0.81-0.96)	-	1.07(1.01-1.14)	-	1.01(0.93-1.10)	-
P-value	<0.005	-	0.017	-	0.777	-
**Marital status**						
Others[b]	1.00	-	1.00	1.00	1.00	-
Married	1.07(0.99-1.16)	-	1.07(1.01-1.14)	1.09(1.02-1.16)	0.94(0.87-1.02)	-
P-value	0.072	-	0.027	0.008	0.160	-
**Education**						
College	1.00	1.00	1.00	1.00	1.00	1.00
High School	0.90(0.81-0.99)	0.94(0.86-1.03)	1.17(1.07-1.28)	1.13(1.04-1.23)	1.02(0.94-1.11)	1.03(0.95-1.12)
Elementary School	0.74(0.67-0.82)	0.75(0.68-0.83)	1.20(1.10-1.31)	1.22(1.12-1.33)	0.75(0.68-0.82)	0.88(0.80-0.96)
Illiterate	0.75(0.63-0.89)	0.68(0.56-0.82)	1.18(1.05-1.32)	1.27(1.12-1.44)	0.67(0.56-0.79)	0.94(0.79-1.11)
P-value	<0.005	<0.005	<0.005	<0.005	<0.005	<0.005
**Area of residence**						
Urban area	1.00	-	1.00	-	1.00	1.00
Rural area	0.84(0.74-0.96)	-	1.12(1.03-1.20)	-	0.71(0.63-0.80)	0.75(0.67-0.85)
P-value	0.009	-	0.005	-	<0.005	<0.005
**Economic Status**						
A-B	1.00	1.00	1.00	1.00	1.00	-
C	0.81(0.74-0.89)	0.87(0.8-0.95)	1.14(1.05-1.24)	1.07(0.99-1.16)	0.92(0.84-1.00)	-
D-E	0.82(0.75-0.90)	0.90(0.82-0.98)	1.18(1.09-1.29)	1.12(1.03-1.21)	0.86(0.78-0.95)	-
P-value	<0.005	<0.005	<0.005	0.030	0.010	-

**Physical Activity**						
Sufficient	1.00	-	1.00	1.00	1.00	-
Insufficient	0.93(0.85-1.02)	-	0.93(0.85-1.02)	1.06(0.98-1.15)	0.93(0.85-1.02)	-
None	0.96(0.88-1.05)	-	1.07(1.01-1.14)	1.09(1.02-1.16)	0.92(0.83-1.02)	-
P-value	0.704	-	0.092	0.016	0.060	-
**Smoking**						
Never	1.00	1.00	1.00	-	1.00	-
Ex-smokers	0.86(0.77-0.97)	0.87(0.79-0.97)	0.95(0.87-1.03)	-	0.81(0.72-0.90)	-
Current	0.77(0.68-0.87)	0.84(0.75-0.95)	1.04(0.96-1.14)	-	0.85(0.75-0.95)	-
P-value	<0.005	<0.005	0.298	-	<0.005	-
**Alcohol intake**						
Abstainer	1.00	-	1.00	-	1.00	1.00
Moderate	1.05(0.97-1.14)	-	1.00(0.93-1.07)	-	1.09(1.00-1.19)	1.02(0.94-1.11)
Binge drinker	0.86(0.75-0.97)	-	1.07(0.98-1.17)	-	1.25(1.14-1.37)	1.12(1.03-1.23)
P-value	0.014	-	0.272	-	<0.005	0.032
**Self-Rated Health**						
Very good/Good	1.00	-	1.00	-	1.00	1.00
Fair	0.93(0.85-1.02)	-	0.98(0.92-1.05)	-	0.85(0.78-0.93)	0.96(0.89-1.05)
Poor/Very poor	0.84(0.69-1.01)	-	0.91(0.79-1.06)	-	0.60(0.46-0.78)	0.71(0.57-0.89)
P-value	0.082	-	0.456	-	<0.005	0.011
**Multimorbidity**						
0 or 1	1.00	-	1.00	-	1.00	-
2	1.04(0.92-1.18)	-	0.94(0.85-1.03)	-	0.79(0.68-0.91)	-
3	1.11(0.94-1.31)	-	0.88(0.76-1.02)	-	0.66(0.54-0.81)	-
4+	1.12(0.95-1.32)	-	0.82(0.70-0.95)	-	0.64(0.51-0.81)	-
P-value	0.334	-	0.015	-	<0.005	-

P-value to the Wald Test.

-: Variables not statistically significant in the model.

[a] Black(a), brown(a), indigenous.

[b] single, divorced, separated, widowed
