# Supplementary material for: Health, lifestyle and sociodemographic characteristics are associated with Brazilian dietary patterns: Brazilian National Health Survey
Source: PLoS One. 2021 Feb 16;16(2):e0247078. doi: 10.1371/journal.pone.0247078 (PMC7886222; doi:10.1371/journal.pone.0247078)
Supplement: S16 Table — Comparison between quartile 1 and quartile 3 for each dietary pattern. (PDF) [file pone.0247078.s016.pdf]

S16 Table. Associations between dietary patterns, lifestyle, health and sociodemographic characteristics in the Northeast Region of Brazil. Comparison between quartile 1 and quartile 3 for each dietary pattern.

DIETARY PATTERNS	HEALTHY		PROTEIN		WESTEN	
**Prevalence Ratio**	**Crude (95%CI)**	**Adjusted (95%CI)**	**Crude (95%CI)**	**Adjusted (95%CI)**	**Crude (95%CI)**	**Adjusted (95%CI)**
**Sample Size (n)**	**9,840**		**9,950**		**10,340**	
**Estimated Population Size (N)**	**21,389,719**		**20,359,248**		**21,401,724**	
**Age groups (years)**						
60+	1.00	1.00	1.00	1.00	1.00	1.00
18-24	0.78(0.67-0.90)	0.54(0.46-0.62)	1.51(1.33-1.71)	1.47(1.28-1.68)	2.47(2.12-2.87)	2.03(1.73-2.38)
25-39	0.89(0.80-0.99)	0.64(0.58-0.72)	1.44(1.29-1.61)	1.38(1.23-1.55)	1.86(1.63-2.13)	1.55(1.34-1.79)
40-59	0.97(0.88-1.07)	0.81(0.74-0.89)	1.29(1.15-1.46)	1.25(1.12-1.41)	1.27(1.10-1.47)	1.13(0.98-1.32)
P-value	<0.005	<0.005	<0.005	<0.005	<0.005	<0.005
**Sex**						
Male	1.00	1.00	1.00	1.00	1.00	-
Female	1.19(1.10-1.28)	1.13(1.05-1.21)	0.66(0.61-0.70)	0.7(0.65-0.75)	0.94(0.87-1.02)	-
P-value	<0.005	<0.005	<0.005	<0.005	0.148	-
**Skin Color/Race**						
White/Yellow	1.00	-	1.00	-	1.00	-
Others[a]	0.89(0.82-0.97)	-	1.03(0.94-1.12)	-	0.90(0.83-0.98)	-
P-value	0.005	-	0.526	-	0.009	-
**Marital status**						
Others[b]	1.00	1.00	1.00	-	1.00	-
Married	1.09(1.01-1.19)	1.09(1.01-1.17)	1.06(0.99-1.13)	-	0.87(0.81-0.94)	-
P-value	0.03	0.03	0.11	-	<0.005	-
**Education**						
College	1.00	1.00	1.00	1.00	1.00	1.00
High School	0.81(0.74-0.88)	0.88(0.81-0.96)	1.28(1.12-1.46)	1.20(1.06-1.37)	1.01(0.92-1.12)	1.01(0.92-1.12)
Elementary School	0.66(0.60-0.73)	0.75(0.68-0.83)	1.31(1.17-1.48)	1.28(1.13-1.45)	0.68(0.61-0.76)	0.83(0.74-0.93)
Illiterate	0.58(0.50-0.67)	0.62(0.53-0.73)	1.15(0.99-1.34)	1.25(1.05-1.49)	0.49(0.42-0.58)	0.72(0.60-0.87)
P-value	<0.005	<0.005	<0.005	<0.005	<0.005	<0.005
**Area of residence**						
Urban area	1.00	1.00	1.00	1.00	1.00	1.00
Rural area	0.68(0.61-0.76)	0.81(0.72-0.92)	1.18(1.07-1.29)	1.11(1.00-1.22)	0.74(0.66-0.84)	0.82(0.73-0.93)
P-value	<0.005	<0.005	<0.005	0.04	<0.005	<0.005
**Economic Status**						
A-B	1.00	1.00	1.00	-	1.00	1.00
C	0.90(0.80-1.01)	1.01(0.92-1.11)	1.13(0.98-1.31)	-	0.78(0.70-0.86)	0.81(0.74-0.89)
D-E	0.68(0.6-0.76)	0.86(0.77-0.97)	1.10(0.95-1.27)	-	0.69(0.62-0.76)	0.83(0.75-0.91)
P-value	<0.005	<0.005	0.25	-	<0.005	<0.005

**Physical Activity**						
Sufficient	1.00	1.00	1.00	-	1.00	-
Insufficient	0.93(0.85-1.02)	0.91(0.83-1.00)	0.93(0.85-1.02)	-	0.93(0.85-1.02)	-
None	0.82(0.75-0.90)	0.81(0.74-0.89)	0.95(0.87-1.04)	-	0.84(0.76-0.92)	-
P-value	<0.005	<0.005	0.49	-	<0.005	-
**Smoking**						
Never	1.00	1.00	1.00	1.00	1.00	-
Ex-smokers	0.91(0.83-1.00)	0.87(0.80-0.95)	1.01(0.93-1.1)	1.07(0.98-1.17)	0.75(0.66-0.85)	-
Current	0.66(0.59-0.74)	0.70(0.63-0.79)	1.22(1.13-1.32)	1.14(1.05-1.23)	0.81(0.71-0.92)	-
P-value	<0.005		<0.005	<0.005	<0.005	-
**Alcohol intake**						
Abstainer	1.00	-	1.00	-	1.00	1.00
Moderate	0.94(0.86-1.02)	-	1.22(1.12-1.32)	-	1.23(1.12-1.34)	1.11(1.02-1.20)
Binge drinker	0.82(0.73-0.93)	-	1.33(1.22-1.45)	-	1.32(1.19-1.47)	1.15(1.05-1.27)
P-value	<0.005	-	<0.005	-	<0.005	<0.005
**Self-Rated Health**						
Very good/Good	1.00	1.00	1.00	-	1.00	-
Fair	0.82(0.75-0.90)	0.79(0.72-0.86)	0.93(0.87-1.00)	-	0.74(0.68-0.81)	-
Poor/Very poor	0.76(0.66-0.87)	0.76(0.65-0.88)	0.75(0.64-0.87)	-	0.62(0.53-0.73)	-
P-value	<0.005	<0.005	<0.005	-	<0.005	-
**Multimorbidity**						
0 or 1	1.00	1.00	1.00	1.00	1.00	-
2	1.14(1.02-1.28)	1.11(1.00-1.24)	0.82(0.73-0.92)	0.92(0.82-1.04)	0.70(0.60-0.80)	-
3	1.14(1.00-1.30)	1.13(0.98-1.30)	0.59(0.48-0.72)	0.70(0.57-0.86)	0.73(0.60-0.89)	-
4+	1.25(1.08-1.46)	1.23(1.05-1.44)	0.61(0.49-0.77)	0.75(0.60-0.93)	0.67(0.53-0.84)	-
P-value	<0.005	0.03	<0.005	<0.005	<0.005	-

P-value to the Wald Test.

-: Variables not statistically significant in the model.

[a] Black(a), brown(a), indigenous.

[b] single, divorced, separated, widowed
