# Supplementary material for: Health, lifestyle and sociodemographic characteristics are associated with Brazilian dietary patterns: Brazilian National Health Survey
Source: PLoS One. 2021 Feb 16;16(2):e0247078. doi: 10.1371/journal.pone.0247078 (PMC7886222; doi:10.1371/journal.pone.0247078)
Supplement: S17 Table — Comparison between quartile 1 and quartile 3 for each dietary pattern. (PDF) [file pone.0247078.s017.pdf]

S17 Table. Associations between dietary patterns, lifestyle, health and sociodemographic characteristics in the North Region of Brazil. Comparison between quartile 1 and quartile 3 for each dietary pattern.

DIETARY PATTERNS	HEALTHY		PROTEIN		WESTEN	
**Prevalence Ratio**	**Crude (95%CI)**	**Adjusted (95%CI)**	**Crude (95%CI)**	**Adjusted (95%CI)**	**Crude (95%CI)**	**Adjusted (95%CI)**
**Sample Size (n)**	**7,334**		**7,222**		**6,471**	
**Estimated Population Size (N)**	**6,523,390**		**6,401,547**		**5,800,089**	
**Age groups (years)**						
60+	1.00	1.00	1.00	1.00	1.00	1.00
18-24	0.70(0.57-0.85)	0.46(0.37-0.57)	1.47(1.16-1.87)	1.46(1.16-1.84)	2.44(2.03-2.93)	2.24(1.86-2.70)
25-39	0.90(0.75-1.07)	0.64(0.53-0.78)	1.44(1.14-1.82)	1.46(1.16-1.83)	2.06(1.73-2.46)	1.91(1.60-2.27)
40-59	0.98(0.82-1.17)	0.82(0.69-0.98)	1.31(1.04-1.66)	1.30(1.04-1.64)	1.43(1.16-1.75)	1.37(1.12-1.68)
P-value	<0.005	<0.005	0.01	<0.005	<0.005	<0.005
**Sex**						
Male	1.00	1.00	1.00	1.00	1.00	-
Female	1.23(1.10-1.37)	1.12(1.01-1.24)	0.85(0.75-0.95)	0.81(0.72-0.90)	0.96(0.87-1.06)	-
P-value	<0.005	0.03	0.01	<0.005	0.42	-
**Skin Color/Race**						
White/Yellow	1.00	1.00	1.00	1.00	1.00	-
Others[a]	0.70(0.61-0.8)	0.82(0.73-0.93)	0.63(0.56-0.72)	0.66(0.59-0.74)	0.91(0.81-1.02)	-
P-value	<0.005	<0.005	<0.005	<0.005	0.11	-
**Marital status**						
Others[b]	1.00	-	1.00	-	1.00	-
Married	0.97(0.87-1.09)	-	0.99(0.87-1.13)	-	0.92(0.83-1.02)	-
P-value	0.66		0.88	-	0.11	-
**Education**						
College	1.00	1.00	1.00	-	1.00	-
High School	0.77(0.67-0.88)	0.86(0.76-0.97)	0.95(0.80-1.11)	-	0.95(0.85-1.06)	-
Elementary School	0.50(0.42-0.58)	0.57(0.49-0.67)	0.74(0.61-0.89)	-	0.64(0.56-0.74)	-
Illiterate	0.49(0.39-0.60)	0.54(0.43-0.68)	0.87(0.68-1.10)	-	0.57(0.46-0.70)	-
P-value	<0.005	<0.005	<0.005	-	<0.005	-
**Area of residence**						
Urban area	1.00	1.00	1.00	1.00	1.00	1.00
Rural area	0.44(0.34-0.57)	0.56(0.43-0.71)	0.61(0.48-0.78)	0.63(0.49-0.80)	0.51(0.41-0.64)	0.54(0.44-0.67)
P-value	<0.005	<0.005	<0.005	<0.005	<0.005	<0.005
**Economic Status**						
A-B	1.00	-	1.00	-	1.00	1.00
C	0.95(0.80-1.12)	-	0.86(0.73-1.01)	-	0.80(0.72-0.89)	0.87(0.78-0.96)
D-E	0.73(0.61-0.87)	-	0.73(0.62-0.86)	-	0.66(0.59-0.75)	0.83(0.75-0.93)
P-value	<0.005	-	<0.005	-	<0.005	<0.005

**Physical Activity**						
Sufficient	1.00	1.00	1.00	-	1.00	-
Insufficient	0.93(0.85-1.02)	0.90(0.78-1.05)	0.93(0.85-1.02)	-	0.93(0.85-1.02)	-
None	0.84(0.74-0.96)	0.81(0.71-0.92)	0.96(0.83-1.11)	-	0.81(0.72-0.90)	-
P-value	0.04	0.01	0.86	-	<0.005	-
**Smoking**						
Never	1.00	1.00	1.00	-	1.00	-
Ex-smokers	0.92(0.76-1.11)	0.92(0.78-1.10)	0.88(0.73-1.06)	-	0.86(0.75-0.98)	-
Current	0.63(0.51-0.77)	0.72(0.59-0.88)	1.17(0.98-1.39)	-	0.89(0.77-1.04)	-
P-value	<0.005	0.01	0.065	-	0.032	-
**Alcohol intake**						
Abstainer	1.00	-	1.00	-	1.00	-
Moderate	0.86(0.75-1.00)	-	1.15(0.99-1.34)	-	1.20(1.07-1.34)	-
Binge drinker	0.90(0.74-1.09)	-	1.28(1.07-1.54)	-	1.26(1.10-1.45)	-
P-value	0.085	-	0.02	-	<0.005	-
**Self-Rated Health**						
Very good/Good	1.00	1.00	1.00	-	1.00	1
Fair	0.84(0.74-0.96)	0.88(0.77-0.99)	0.94(0.82-1.07)	-	0.75(0.67-0.84)	0.88(0.79-0.98)
Poor/Very poor	0.59(0.45-0.78)	0.63(0.47-0.84)	0.96(0.71-1.31)	-	0.60(0.48-0.77)	0.83(0.66-1.05)
P-value	<0.005	<0.005	0.627	-	<0.005	0.04
**Multimorbidity**						
0 or 1	1.00	1.00	1.00	-	1.00	-
2	1.02(0.83-1.25)	0.99(0.80-1.22)	0.95(0.79-1.14)	-	0.85(0.72-1.00)	-
3	1.48(1.20-1.84)	1.39(1.14-1.70)	0.78(0.60-1.02)	-	0.77(0.60-0.99)	-
4+	1.59(1.16-2.17)	1.44(1.10-1.87)	0.91(0.58-1.42)	-	0.72(0.49-1.05)	-
P-value	<0.005	<0.005	0.306	-	0.03	-

P-value to the Wald Test.

-: Variables not statistically significant in the model.

[a] Black(a), brown(a), indigenous.

[b] single, divorced, separated, widowed
